# Supplementary material for: Proteomic studies of VEGFR2 in human placentas reveal protein associations with preeclampsia, diabetes, gravidity, and labor
Source: Cell Commun Signal. 2024 Apr 9;22:221. doi: 10.1186/s12964-024-01567-0 (PMC11003095; doi:10.1186/s12964-024-01567-0)
Supplement: Supplementary file 1 — Supplementary Material 1. [file 12964_2024_1567_MOESM1_ESM.zip › Ho S et al Supplementary Tables with corrected manuscript Title 3-25-24_ESM.pdf]

**List of Supplementary Tables and Video**

# Proteomic studies of VEGFR2 in human placentas reveal protein associations with preeclampsia, diabetes, gravidity, and labor

**Shannon J. Ho[1,10], Dale Chaput[2,10], Rachel G. Sinkey[1], Amanda H. Garces[3], Erika P. New[1], Maja Okuka[1], Peng Sang[4], Sefa Arlier[1], Nihan Semerci[1], Thora S. Steffensen[5], Thomas J. Rutherford[1,6], Angel E. Alsina[7], Jianfeng Cai[4], Matthew L. Anderson[1,6], Ronald R. Magness[1], Vladimir N. Uversky[8], Derek A. T. Cummings[9] & John C. M. Tsibris[1,8] ✉**

[1]Department of Obstetrics and Gynecology, University of South Florida, Tampa, Florida, USA. [2]Department of Cell Biology, Microbiology and Molecular Biology, University of South Florida, Tampa, Florida, USA. [3]Lisa Muma Weitz Microscopy Laboratory, University of South Florida, USA. [4]Department of Chemistry, University of South Florida, Tampa, Florida, USA. [5]Department of Pathology, Tampa General Hospital, Tampa, Florida, USA. [6]Cancer Center, Tampa General Hospital, Tampa, Florida, USA. [7]Transplant Surgery Center, Tampa General Hospital, Tampa, Florida, USA. [8]Department of Molecular Medicine, University of South Florida, Tampa, Florida, USA. [9]Department of Biology and Emerging Pathogens Institute, University of Florida, Gainesville, FL, USA. [10]These authors contributed equally. ✉e-mail: tsibris@usf.edu

Supplementary Table 1. Proteins identified in VEGFR2 immunoprecipitations.
Supplementary Table 2. Proteins identified in MDMX immunoprecipitations (IP).
Supplementary Table 3. Proteins identified in PICALM immunoprecipitations.
Supplementary Table 4. Protein levels of MDMX (A), PICALM (B), OT-R (C), and V1aR (D) in 44 placental extracts.
Supplementary Table 5. Regression coefficients from univariable analysis of MDMX protein band intensity (relative to internal reference sample Q1).

Supplementary Table 6. Regression coefficients from univariable analysis of PICALM protein band intensity (relative to internal reference sample Q1).

Supplementary Table 7. Regression coefficients from univariable analysis of OT-R protein band intensity (relative to internal reference sample T1).

Supplementary Table 8. Regression coefficients from univariable analysis of V1aR protein band intensity (relative to internal reference sample T1).

Supplementary Table 9. Regression coefficients from multivariable analysis of MDMX protein band intensity (relative to internal reference sample Q1).

Supplementary Table 10. Regression coefficients from multivariable analysis of PICALM protein band intensity (relative to internal reference sample Q1).

Supplementary Table 11. Regression coefficients from multivariable analysis of OT-R protein band intensity (relative to internal reference sample T1).

Supplementary Table 12. Regression coefficients from bootstrapped replicates of univariable analysis of MDMX protein band intensity (relative to internal reference sample Q1).

Supplementary Table 13. Regression coefficients from bootstrapped replicates of univariable analysis of PICALM protein band intensity (relative to internal reference sample Q1).

Supplementary Table 14. Regression coefficients from bootstrapped replicates of univariable analysis of OT-R protein band intensity (relative to internal reference sample T1).

Supplementary Table 15. Regression coefficients from bootstrapped replicates univariable analysis of V1aR protein band intensity (relative to internal reference sample T1).

Supplementary Table 16. Regression coefficients from bootstrapped replicates of multivariable analysis of MDMX protein band intensity (relative to internal reference sample Q1).

Supplementary Table 17. Regression coefficients from bootstrapped replicates of multivariable analysis of PICALM protein band intensity (relative to internal reference sample Q1).

Supplementary Table 18. Regression coefficients from bootstrapped replicates of multivariable analysis of OT-R protein band intensity (relative to internal reference sample T1).

Supplementary Table 19. Autoantigens listed by Neiman et al. [52] among the proteins in VEGFR2, MDMX, and PICALM immunoprecipitations (IP).

Supplementary Table 20. Patient demographics.

Supplementary Video. The chorionic villi. Colocalization of VEGFR2 (red) and PDC-E2 (green) in endothelial cells of the villous vasculature of a normotensive, non-diabetic patient is shown in the video obtained from reconstructed stacked images of whole mount immunofluorescence. Nuclei were stained blue with DAPI.

**Supplemental Table 1**
**Proteins identified in VEGFR2 immunoprecipitations.**
Placental extracts O4, O5, U1, and O7 were immunoprecipitated in one experiment.

| Majority Protein ID | Protein | Gene | Total Intensity | Intensity VEGFR2 O4 | Intensity VEGFR2 O5 | Intensity VEGFR2 U1 | Intensity VEGFR2 O7 | Peptides |
|---|---|---|---|---|---|---|---|---|
| P01857 | Ig gamma-1 chain C region | IGHG1 | 24,993,000,000 | 8,554,400,000 | 4,872,000,000 | 4,017,600,000 | 7,549,400,000 | 16 |
| O15151-5 | Protein Mdm4 | MDM4 | 14,682,000,000 | 14,638,000,000 | 0 | 21,714,000 | 22,289,000 | 14 |
| P01834 | Ig kappa chain C region | IGKC | 11,450,000,000 | 1,978,700,000 | 1,363,300,000 | 2,438,900,000 | 5,668,800,000 | 7 |
| A0A0B4J231 | Immunoglobulin lambda-like polypeptide 5;Ig lam | IGLL5 | 10,336,000,000 | 1,336,300,000 | 2,654,400,000 | 2,053,600,000 | 4,291,800,000 | 4 |
| P01871 | Ig mu chain C region | IGHM | 4,544,300,000 | 2,132,500,000 | 923,280,000 | 706,680,000 | 781,900,000 | 16 |
| P20073-2 | Annexin A7 | ANXA7 | 3,045,300,000 | 1,631,900,000 | 402,390,000 | 522,590,000 | 488,400,000 | 19 |
| P01624 | Ig kappa chain V-III region POM | IGKV3D-15 | 2,885,700,000 | 707,630,000 | 775,800,000 | 20,798,000 | 1,381,500,000 | 2 |
| A0A286YES1 | Ig gamma-3 chain C region | IGHG3 | 2,266,900,000 | 1,322,900,000 | 493,150,000 | 239,480,000 | 211,400,000 | 14 |
| Q9Y6R7 | IgGFc-binding protein | FCGBP | 1,815,700,000 | 1,201,900,000 | 310,570,000 | 246,590,000 | 56,630,000 | 50 |
| J3KT55 | Protein-tyrosine-phosphatase;Receptor-type tyro | PTPRM | 1,604,700,000 | 511,350,000 | 129,960,000 | 380,380,000 | 582,970,000 | 1 |
| P01701 | Ig lambda chain V-I region NEW | IGLV1-51 | 1,311,400,000 | 193,890,000 | 242,130,000 | 234,850,000 | 640,570,000 | 3 |
| P50995-2 | Annexin A11 | ANXA11 | 984,170,000 | 401,780,000 | 125,800,000 | 283,580,000 | 173,010,000 | 18 |
| P01583 | Ig kappa chain V-I region AU | IGKV1D-33 | 948,420,000 | 223,440,000 | 220,510,000 | 138,140,000 | 366,330,000 | 2 |
| P01709 | Ig lambda chain V-II region MGC; | IGLV2-8 | 640,270,000 | 48,724,000 | 120,690,000 | 169,210,000 | 301,660,000 | 2 |
| P05783 | Keratin, type I cytoskeletal 18 | KRT18 | 610,030,000 | 342,270,000 | 114,070,000 | 75,571,000 | 78,111,000 | 19 |
| P35968 | Vascular endothelial growth factor receptor 2 | KDR | 571,130,000 | 132,550,000 | 167,190,000 | 146,080,000 | 125,310,000 | 28 |
| A0A286YEY4 | Ig gamma-2 chain C region | IGHG2 | 460,180,000 | 213,440,000 | 63,593,000 | 99,658,000 | 83,492,000 | 13 |
| A0A286YEY1 | Ig alpha-1 chain C region | IGHA1 | 403,780,000 | 211,760,000 | 52,913,000 | 85,992,000 | 53,116,000 | 6 |
| Q8WZ42 | Titin | TTN | 396,070,000 | 215,440,000 | 31,243,000 | 97,005,000 | 52,381,000 | 2 |
| A0A075B6K4 | Ig lambda chain V-IV region Bau | IGLV3-10 | 353,630,000 | 0 | 105,060,000 | 0 | 248,570,000 | 1 |
| P01602 | Ig kappa chain V-I region HK102 | IGKV1-5 | 352,740,000 | 23,518,000 | 98,394,000 | 83,336,000 | 147,490,000 | 1 |
| P07355 | Annexin A2;Annexin;Putative annexin A2-like prc | ANXA2 | 310,810,000 | 50,471,000 | 50,738,000 | 48,427,000 | 161,170,000 | 9 |
| P0DP08 | Ig heavy chain V-II region NEWM;Ig heavy chain | IGHV4-61 | 304,360,000 | 139,200,000 | 44,252,000 | 64,988,000 | 55,917,000 | 2 |
| A0A4W8ZXM2 | Immunoglobulin heavy variable 3-72 | IGHV3-72 | 293,310,000 | 149,140,000 | 29,272,000 | 50,152,000 | 64,749,000 | 3 |
| A0A140T8W4 | Ras/Rap GTPase-activating protein SynGAP | SYNGAP1 | 288,030,000 | 44,004,000 | 63,931,000 | 66,499,000 | 113,600,000 | 1 |
| P01619 | Ig kappa chain V-III region B6 | IGKV3D-20 | 270,720,000 | 61,855,000 | 116,270,000 | 75,359,000 | 17,241,000 | 3 |
| A0A0C4DH42 | Ig heavy chain V-III region BUT;Ig heavy chain V | IGHV3-66 | 262,750,000 | 165,010,000 | 39,894,000 | 44,120,000 | 13,734,000 | 3 |
| A0A087WW87 | Ig kappa chain V-II region FR;Ig kappa chain V-II | IGKV2-40 | 253,710,000 | 36,751,000 | 64,720,000 | 43,474,000 | 108,760,000 | 3 |
| P59666 | Neutrophil defensin 3;HP 3-56;Neutrophil defens | DEFA3 | 216,520,000 | 69,150,000 | 64,470,000 | 3,576,200 | 79,326,000 | 3 |
| P69905 | Hemoglobin subunit alpha | HBA1 | 215,170,000 | 0 | 0 | 7,679,400 | 207,490,000 | 3 |
| P68871 | Hemoglobin subunit beta;LVV-hemorphin-7;Spin | HBB | 212,010,000 | 31,855,000 | 0 | 8,761,200 | 171,400,000 | 5 |
| A0A2R8Y804 | Catenin beta-1 | CTNNB1 | 209,290,000 | 166,480,000 | 20,293,000 | 22,516,000 | 0 | 1 |
| H0Y2X5 | Aldehyde dehydrogenase family 1 member A3 | ALDH1A3 | 208,040,000 | 81,161,000 | 28,288,000 | 92,767,000 | 5,824,900 | 1 |
| P0DOY3 | Ig lambda-6 chain C region;Ig lambda-7 chain C | IGLC6 | 206,310,000 | 71,942,000 | 14,393,000 | 49,459,000 | 70,513,000 | 3 |
| P17931 | Galectin-3;Galectin | LGALS3 | 177,750,000 | 8,812,900 | 12,846,000 | 37,357,000 | 118,730,000 | 4 |
| P60709 | Actin, cytoplasmic 1;Actin, cytoplasmic 1, N-term | ACTB | 169,720,000 | 77,252,000 | 11,135,000 | 41,447,000 | 39,882,000 | 7 |
| P0DP03 | Ig heavy chain V-III region CAM;Ig heavy chain V | IGHV3-23 | 161,210,000 | 84,420,000 | 37,310,000 | 16,539,000 | 22,945,000 | 3 |
| P08670 | Vimentin | VIM | 138,160,000 | 72,126,000 | 18,191,000 | 31,138,000 | 16,707,000 | 14 |
| A0A0A0MRZ8 | Ig kappa chain V-III region VG | IGKV3D-11 | 111,170,000 | 34,909,000 | 76,260,000 | 0 | 0 | 1 |
| A0A075B6K5 | Ig lambda chain V-III region LOI | IGLV3-9 | 96,087,000 | 25,060,000 | 23,281,000 | 14,993,000 | 32,753,000 | 1 |
| A0A286YFJ8 | Ig gamma-4 chain C region | IGHG4 | 87,485,000 | 47,260,000 | 30,192,000 | 1,565,900 | 8,467,600 | 9 |
| A0A075B6Z5 | T cell receptor alpha joining 4 | TRAJ4 | 81,903,000 | 16,074,000 | 21,003,000 | 0 | 44,826,000 | 1 |
| Q96Q89-4 | Kinesin-like protein KIF20B | KIF20B | 80,885,000 | 35,045,000 | 8,063,900 | 37,776,000 | 0 | 1 |
| P31942-3 | Heterogeneous nuclear ribonucleoprotein H3 | HNRNPH3 | 80,200,000 | 14,536,000 | 9,591,700 | 10,848,000 | 45,224,000 | 4 |
| P0DP01 | Ig heavy chain V-I region HG3;Ig heavy chain V-I | IGHV1-3 | 75,366,000 | 33,904,000 | 12,319,000 | 16,686,000 | 12,457,000 | 1 |
| C9JA05 | Immunoglobulin J chain | JCHAIN | 74,956,000 | 6,239,800 | 24,681,000 | 8,971,600 | 35,063,000 | 1 |
| A6NJ08 | Putative methyl-CpG-binding domain protein 3-lik | MBD3L5 | 72,585,000 | 0 | 0 | 72,585,000 | 0 | 1 |
| O43866 | CD5 antigen-like | CD5L | 67,251,000 | 6,765,200 | 12,049,000 | 0 | 48,437,000 | 3 |
| A0A075B6H9 | Immunoglobulin lambda variable 4-69 | IGLV4-69 | 66,271,000 | 0 | 23,139,000 | 15,467,000 | 27,664,000 | 1 |
| A0A0C4DH31 | Ig heavy chain V-I region V35 | IGHV1-18 | 66,003,000 | 33,480,000 | 6,768,000 | 14,275,000 | 11,480,000 | 2 |
| Q8IZ41 | Ras and EF-hand domain-containing protein | RASEF | 63,614,000 | 0 | 0 | 0 | 63,614,000 | 1 |
| Q5JTQ6 | Alpha-catulin | CTNNAL1 | 57,823,000 | 0 | 0 | 0 | 57,823,000 | 1 |
| S4R460 | Immunoglobulin heavy variable 3/OR16-9 | IGHV3OR16-9 | 55,031,000 | 38,287,000 | 3,882,000 | 8,163,400 | 4,697,800 | 3 |
| P31943 | Heterogeneous nuclear ribonucleoprotein H;Hete | HNRNPH1 | 53,003,000 | 27,450,000 | 6,928,200 | 12,581,000 | 6,044,000 | 8 |
| P01700 | Ig lambda chain V-I region HA | IGLV1-47 | 52,593,000 | 4,428,100 | 9,274,400 | 10,578,000 | 28,313,000 | 2 |
| P69892 | Hemoglobin subunit gamma-2;Hemoglobin subur | HBG2 | 51,210,000 | 4,832,700 | 6,432,400 | 4,039,100 | 35,906,000 | 2 |
| Q01469 | Fatty acid-binding protein, epidermal | FABP5 | 49,218,000 | 9,923,200 | 8,054,700 | 6,476,700 | 24,764,000 | 1 |
| A0A0C4DH38 | Immunoglobulin heavy variable 5-51 | IGHV5-51 | 47,777,000 | 21,306,000 | 9,176,600 | 9,526,200 | 7,768,200 | 2 |
| P06312 | Ig kappa chain V-IV region | IGKV4-1 | 45,342,000 | 3,210,900 | 8,777,500 | 9,179,900 | 24,174,000 | 2 |
| A0A075B7B8 | Immunoglobulin heavy variable 3/OR16-12 | IGHV3OR16-12 | 42,463,000 | 23,390,000 | 6,964,700 | 8,836,000 | 3,272,300 | 2 |
| D6RF44 | Heterogeneous nuclear ribonucleoprotein D0 | HNRNPD | 42,167,000 | 25,423,000 | 6,531,800 | 3,981,200 | 6,231,100 | 2 |
| P12314 | High affinity immunoglobulin gamma Fc receptor | FCGR1A | 41,035,000 | 21,744,000 | 3,306,900 | 13,766,000 | 2,217,200 | 4 |
| A0A075B6I0 | Immunoglobulin lambda variable 8-61 | IGLV8-61 | 36,318,000 | 0 | 15,369,000 | 0 | 20,949,000 | 1 |
| P01023 | Alpha-2-macroglobulin | A2M | 34,428,000 | 24,712,000 | 0 | 5,408,900 | 4,307,000 | 9 |
| M0R1R1 | Serine/threonine-protein kinase PAK 4 | PAK4 | 33,472,000 | 0 | 0 | 33,472,000 | 0 | 1 |
| A0A1C7CYZ1 | Mitogen-activated protein kinase 15 | MAPK15 | 33,062,000 | 6,944,400 | 14,185,000 | 7,347,000 | 4,586,000 | 1 |
| A0A0C4DGL8 | Haptoglobin;Haptoglobin alpha chain;Haptoglobi | HP | 32,080,000 | 0 | 0 | 0 | 32,080,000 | 2 |
| P05109 | Protein S100-A8;Protein S100-A8, N-terminally p | S100A8 | 29,241,000 | 14,931,000 | 0 | 10,002,000 | 4,308,000 | 2 |
| Q03135 | Caveolin-1;Caveolin | CAV1 | 28,128,000 | 0 | 0 | 0 | 28,128,000 | 2 |
| P01714 | Ig lambda chain V-III region SH | IGLV3-19 | 26,041,000 | 4,373,400 | 0 | 0 | 21,668,000 | 1 |
| P19474 | E3 ubiquitin-protein ligase TRIM21 | TRIM21 | 25,255,000 | 18,886,000 | 4,813,800 | 1,555,300 | 0 | 4 |
| P62979 | Ubiquitin-40S ribosomal protein S27a;Ubiquitin;4 | RPS27A | 25,055,000 | 1,656,300 | 2,801,700 | 7,174,300 | 13,423,000 | 2 |
| P81605 | Dermcidin;Survival-promoting peptide;DCD-1 | DCD | 22,842,000 | 12,716,000 | 3,502,900 | 0 | 6,623,700 | 2 |
| P52594-2 | Arf-GAP domain and FG repeat-containing protei | AGFG1 | 22,167,000 | 16,991,000 | 2,315,700 | 2,860,400 | 0 | 1 |
| A0A0C4DH35 | Probable non-functional immunoglobulin | IGHV3-35 | 20,964,000 | 12,648,000 | 3,344,000 | 2,850,000 | 2,122,100 | 1 |
| Q86VF2-5 | Isoform 5 of Immunoglobulin-like | IGFN1 | 20,453,000 | 0 | 0 | 0 | 20,453,000 | 1 |
| K7EJT5 | 60S ribosomal protein L22 | RPL22 | 18,553,000 | 4,268,800 | 0 | 0 | 14,284,000 | 2 |
| A0A0A0MSV6 | Complement C1q subcomponent subunit B | C1QB | 18,258,000 | 0 | 6,523,400 | 0 | 11,734,000 | 2 |
| A0A075B6K0 | Ig lambda chain V-IV region Hil;Ig lambda chain V | IGLV3-16 | 18,172,000 | 0 | 4,095,500 | 3,407,600 | 10,669,000 | 1 |
| P06702 | Protein S100-A9 | S100A9 | 17,656,000 | 10,525,000 | 0 | 7,131,300 | 0 | 2 |
| F8W0I9 | Microspherule protein 1 | MCRS1 | 16,973,000 | 16,973,000 | 0 | 0 | 0 | 1 |
| P48741 | Putative heat shock 70 kDa protein 7;Heat shock | HSPA7 | 15,835,000 | 7,272,600 | 1,230,000 | 4,196,900 | 3,135,800 | 2 |

| | | | | | | | | |
|---|---|---|---|---|---|---|---|---|
| P04430 | Ig kappa chain V-I region BAN | IGKV1-16 | 15,155,000 | 0 | 1,349,600 | 3,349,700 | 10,456,000 | 1 |
| O75594 | Peptidoglycan recognition protein 1 | PGLYRP1 | 14,595,000 | 2,156,900 | 2,885,000 | 0 | 9,552,700 | 1 |
| P01599 | Ig kappa chain V-I region Gal | IGKV1-17 | 14,133,000 | 1,638,200 | 1,801,900 | 5,586,300 | 5,106,400 | 1 |
| A0A6Q8PHQ9 | Prelamin-A/C;Lamin-A/C | LMNA | 13,477,000 | 10,473,000 | 0 | 990,760 | 2,014,100 | 3 |
| F8WCU1 | Coiled-coil domain-containing protein 150 | CCDC150 | 13,165,000 | 13,165,000 | 0 | 0 | 0 | 1 |
| P11021 | 78 kDa glucose-regulated protein | HSPA5 | 12,097,000 | 12,097,000 | 0 | 0 | 0 | 3 |
| P10515 | Dihydrolipoyllysine-residue acetyltransferase cor | DLAT | 11,577,000 | 0 | 0 | 0 | 11,577,000 | 2 |
| A0A0C4DH73 | Ig kappa chain V-I region Daudi;Ig kappa chain V | IGKV1-12 | 11,157,000 | 4,228,600 | 3,358,000 | 0 | 3,570,100 | 1 |
| Q02413 | Desmoglein-1 | DSG1 | 11,044,000 | 6,539,700 | 4,504,100 | 0 | 0 | 4 |
| A0A2R8Y851 | 40S ribosomal protein S29 | RPS29 | 10,819,000 | 0 | 0 | 0 | 10,819,000 | 1 |
| E9PHT9 | Annexin;Annexin A5 | ANXA5 | 10,757,000 | 0 | 0 | 0 | 10,757,000 | 2 |
| P01766 | Ig heavy chain V-III region BRO | IGHV3-13 | 9,742,100 | 6,872,500 | 2,869,600 | 0 | 0 | 3 |
| I3L0Q1 | CREB-binding protein;Histone acetyltransferase | CREBBP | 9,408,100 | 9,408,100 | 0 | 0 | 0 | 2 |
| A0A0A0MT36 | Immunoglobulin kappa variable 6D-21 | IGKV6D-21 | 9,327,900 | 0 | 0 | 0 | 9,327,900 | 1 |
| P35030-5 | Trypsin-3 | PRSS3 | 8,538,700 | 4,010,200 | 0 | 0 | 4,528,500 | 1 |
| P02747 | Complement C1q subcomponent subunit C | C1QC | 8,466,800 | 0 | 6,587,500 | 1,879,200 | 0 | 2 |
| A0A0B4J1V0 | Immunoglobulin heavy variable 3-15 | IGHV3-15 | 8,461,900 | 2,460,400 | 6,001,500 | 0 | 0 | 2 |
| C9JD14 | Guanine nucleotide-binding protein G(I)/G(S)/G(T | GNB4 | 8,022,200 | 0 | 0 | 0 | 8,022,200 | 1 |
| A0A0C4DH68 | Immunoglobulin kappa variable 2-24 | IGKV2-24 | 7,515,800 | 7,515,800 | 0 | 0 | 0 | 2 |
| Q5T3N1 | Annexin;Annexin A1 | ANXA1 | 7,426,700 | 1,331,300 | 0 | 803,800 | 5,291,600 | 2 |
| A0A0A0MRA5 | Heterogeneous nuclear ribonucleoprotein U-like | HNRNPUL1 | 6,653,000 | 6,653,000 | 0 | 0 | 0 | 3 |
| P0DP04 | Ig heavy chain V-III region DOB | IGHV3-43D | 6,600,800 | 0 | 3,028,100 | 3,572,700 | 0 | 3 |
| P02675 | Fibrinogen beta chain;Fibrinopeptide B;Fibrinoge | FGB | 6,128,300 | 6,128,300 | 0 | 0 | 0 | 2 |
| Q9NZT1 | Calmodulin-like protein 5 | CALML5 | 6,023,300 | 1,202,800 | 0 | 4,408,400 | 412,040 | 3 |
| A0A0C4DH67 | Immunoglobulin kappa variable 1-8 | IGKV1-8 | 5,877,100 | 2,421,000 | 3,456,100 | 0 | 0 | 1 |
| A0A2R8YD12 | Serpin B6 | SERPINB6 | 5,710,400 | 5,710,400 | 0 | 0 | 0 | 1 |
| A0A0A0MRQ5 | Peroxiredoxin-2;Peroxiredoxin-1 | PRDX1 | 5,706,700 | 0 | 0 | 2,361,100 | 3,345,600 | 1 |
| Q5VVL7 | Lipoamide acyltransferase component of branche | DBT | 5,612,200 | 3,395,200 | 0 | 2,217,000 | 0 | 1 |
| P06310 | Ig kappa chain V-II region RPMI 6410 | IGKV2-30 | 5,336,000 | 0 | 0 | 2,017,100 | 3,318,900 | 2 |
| P50991 | T-complex protein 1 subunit delta | CCT4 | 4,474,800 | 0 | 0 | 0 | 4,474,800 | 1 |
| A0A0C4DH36 | Probable non-functional immunoglobulin heavy v | IGHV3-38 | 4,238,600 | 0 | 4,238,600 | 0 | 0 | 2 |
| P47929 | Galectin-7 | LGALS7 | 4,195,800 | 0 | 0 | 1,755,800 | 2,440,000 | 3 |
| B4E1S2 | Annexin;Annexin A4 | ANXA4 | 4,022,500 | 0 | 0 | 0 | 4,022,500 | 1 |
| E7ETU5 | RNA-binding motif, single-stranded-interacting pr | RBMS1 | 3,858,200 | 2,150,500 | 0 | 1,707,700 | 0 | 2 |
| I3L1P8 | Mitochondrial 2-oxoglutarate/malate carrier prote | SLC25A11 | 3,820,000 | 0 | 0 | 0 | 3,820,000 | 1 |
| Q71U36-2 | Tubulin alpha-1A chain;Tubulin alpha-1B chain;T | TUBA1A | 3,782,000 | 3,782,000 | 0 | 0 | 0 | 2 |
| D6R9P3 | Heterogeneous nuclear ribonucleoprotein A/B | HNRNPAB | 3,571,200 | 3,120,400 | 0 | 450,800 | 0 | 2 |
| E9PJT1 | Phosphatidylinositol-binding clathrin assembly pr | PICALM | 3,561,800 | 3,561,800 | 0 | 0 | 0 | 1 |
| A0A0B4J1V6 | Immunoglobulin heavy variable 3-73 | IGHV3-73 | 3,460,100 | 0 | 1,501,600 | 1,958,600 | 0 | 3 |
| P14923 | Junction plakoglobin | JUP | 3,261,700 | 2,178,900 | 0 | 0 | 1,082,900 | 3 |
| Q5T749 | Keratinocyte proline-rich protein | KPRP | 3,196,400 | 3,196,400 | 0 | 0 | 0 | 2 |
| P15924 | Desmoplakin | DSP | 2,976,300 | 0 | 2,479,300 | 0 | 497,000 | 3 |
| E9PKD2 | Nuclear pore complex protein Nup214 | NUP214 | 2,854,500 | 1,488,400 | 1,366,100 | 0 | 0 | 1 |
| B3KV94 | Lysine-specific demethylase 5B | KDM5B | 2,598,600 | 0 | 2,598,600 | 0 | 0 | 1 |
| P78406 | mRNA export factor | RAE1 | 2,408,100 | 2,408,100 | 0 | 0 | 0 | 1 |
| P17661 | Desmin | DES | 2,140,500 | 0 | 0 | 1,047,300 | 1,093,200 | 4 |
| P31994-5 | Low affinity immunoglobulin gamma Fc | FCGR2B | 2,042,300 | 0 | 2,042,300 | 0 | 0 | 1 |
| A0A087WT15 | N-acetylglucosaminyldiphosphodolichol | ALG13 | 1,878,000 | 0 | 1,878,000 | 0 | 0 | 1 |
| P31944 | Caspase-14;Caspase-14 subunit p17, mature for | CASP14 | 880,720 | 0 | 880,720 | 0 | 0 | 1 |
| A0A3B3ISA6 | Complement C4-A;Complement C4 beta chain;C | C4B | 664,880 | 0 | 0 | 0 | 664,880 | 1 |

**Supplemental Table 2**
**Proteins identified in MDMX immunoprecipitations (IP).**
Protein extracts O1, O4, O6, Q1, Q4, and R4 were immunoprecipitated in one experiment.

| Unique to MDMX IP | Common to all IP | Majority protein ID | Protein | Gene | Total Intensity | Intensity MDMX 01 | Intensity MDMX 04 | Intensity MDMX 06 | Intensity MDMX Q1 | Intensity MDMX Q4 | Intensity MDMX R4 | Peptides |
|---|---|---|---|---|---|---|---|---|---|---|---|---|
| | | P01857 | Ig gamma-1 chain C region | IGHG1 | 172,120,000,000 | 22,238,000,000 | 2,879,300,000 | 4,953,400,000 | 85,828,000,000 | 8,388,500,000 | 47,830,000,000 | 17 |
| | Yes | P68871 | Hemoglobin subunit beta | HBB | 49,360,000,000 | 2,016,400,000 | 36,158,000,000 | 924,450,000 | 902,880,000 | 6,065,100,000 | 3,293,300,000 | 14 |
| | Yes | P69905 | Hemoglobin subunit alpha | HBA1 | 46,601,000,000 | 3,529,000,000 | 26,425,000,000 | 1,616,500,000 | 1,416,300,000 | 6,485,900,000 | 7,128,000,000 | 15 |
| | | P20073-2 | Annexin A7 | ANXA7 | 25,375,000,000 | 5,386,800,000 | 1,897,400,000 | 6,836,600,000 | 2,658,100,000 | 3,368,800,000 | 5,226,900,000 | 31 |
| | Yes | Q9Y6R7 | IgGFc-binding protein | FCGBP | 19,471,000,000 | 6,976,500,000 | 5,284,100,000 | 2,450,000,000 | 1,183,900,000 | 470,780,000 | 3,105,900,000 | 75 |
| | | P81605 | Dermcidin;Survival-promoting peptide;DCD-1 | DCD | 11,575,000,000 | 2,289,500,000 | 1,337,700,000 | 2,158,100,000 | 2,832,500,000 | 985,260,000 | 1,971,800,000 | 9 |
| Yes | | P50995 | Annexin A11 | ANXA11 | 11,312,000,000 | 478,090,000 | 1,687,700,000 | 1,675,600,000 | 1,407,600,000 | 3,235,200,000 | 2,827,400,000 | 22 |
| | Yes | P01871 | Ig mu chain C region | IGHM | 10,253,000,000 | 2,115,600,000 | 3,548,100,000 | 788,140,000 | 926,840,000 | 1,253,200,000 | 1,620,700,000 | 18 |
| | | P63261 | Actin, cytoplasmic 2 | ACTG1 | 7,787,800,000 | 669,640,000 | 2,068,500,000 | 955,900,000 | 645,430,000 | 2,563,700,000 | 884,630,000 | 16 |
| | Yes | P69892 | Hemoglobin subunit gamma-2 | HBG2 | 7,148,400,000 | 1,386,800,000 | 780,010,000 | 795,220,000 | 661,950,000 | 460,580,000 | 3,063,800,000 | 12 |
| Yes | | P55072 | Transitional endoplasmic reticulum ATPase | VCP | 4,620,000,000 | 185,550,000 | 758,330,000 | 1,565,000,000 | 411,970,000 | 553,090,000 | 1,146,000,000 | 39 |
| | Yes | P07355 | Annexin A2;Annexin;Putative annexin A2-like | ANXA2 | 4,497,800,000 | 586,000,000 | 417,330,000 | 1,170,600,000 | 722,980,000 | 573,440,000 | 1,027,400,000 | 24 |
| Yes | | P15924 | Desmoplakin | DSP | 4,032,200,000 | 470,760,000 | 397,430,000 | 777,640,000 | 503,940,000 | 1,011,000,000 | 871,340,000 | 71 |
| | Yes | Q02413 | Desmoglein-1 | DSG1 | 3,842,500,000 | 671,480,000 | 532,380,000 | 484,300,000 | 578,340,000 | 684,390,000 | 891,590,000 | 23 |
| | Yes | P01834 | Ig kappa chain C region | IGKC | 3,261,700,000 | 842,260,000 | 1,005,100,000 | 115,500,000 | 198,480,000 | 706,400,000 | 393,970,000 | 8 |
| | | P08670 | Vimentin | VIM | 3,157,700,000 | 489,550,000 | 337,990,000 | 734,410,000 | 251,720,000 | 392,940,000 | 951,130,000 | 25 |
| Yes | | A0A0C4DH90 | Ig kappa chain V-III region POM | IGKV3OR2-268 | 2,570,200,000 | 1,897,100,000 | 0 | 13,227,000 | 0 | 225,870,000 | 434,010,000 | 1 |
| Yes | | V9HW50 | Alcohol dehydrogenase 1B | HEL-S-117 | 2,525,500,000 | 0 | 2,445,000,000 | 0 | 0 | 80,447,000 | 0 | 17 |
| | Yes | P17931 | Galectin-3;Galectin | LGALS3 | 2,429,700,000 | 1,214,100,000 | 169,570,000 | 378,710,000 | 177,170,000 | 77,588,000 | 412,490,000 | 8 |
| Yes | | Q08554-2 | Desmocollin-1 | DSC1 | 2,289,200,000 | 409,040,000 | 260,520,000 | 496,130,000 | 269,620,000 | 267,980,000 | 585,960,000 | 11 |
| | Yes | P0DOY3 | Ig lambda-1 chain C regions | IGLC1 | 2,060,500,000 | 902,940,000 | 302,390,000 | 106,470,000 | 55,564,000 | 332,740,000 | 360,390,000 | 6 |
| | Yes | P14923 | Junction plakoglobin | JUP | 1,729,100,000 | 212,940,000 | 113,640,000 | 343,050,000 | 269,710,000 | 404,730,000 | 385,000,000 | 15 |
| Yes | | Q8N5G2-2 | Macoilin | MACO1 | 1,581,700,000 | 99,294,000 | 476,890,000 | 430,010,000 | 308,790,000 | 0 | 266,730,000 | 2 |
| | | Q5T749 | Keratinocyte proline-rich protein | KPRP | 1,575,500,000 | 330,330,000 | 141,660,000 | 226,220,000 | 285,640,000 | 313,090,000 | 278,590,000 | 12 |
| | Yes | P19474 | E3 ubiquitin-protein ligase TRIM21 | TRIM21 | 1,493,600,000 | 373,480,000 | 221,830,000 | 137,660,000 | 252,360,000 | 260,040,000 | 248,270,000 | 10 |
| | Yes | Q9NZT1 | Calmodulin-like protein 5 | CALML5 | 1,415,300,000 | 243,820,000 | 182,070,000 | 222,180,000 | 153,060,000 | 70,118,000 | 544,030,000 | 5 |
| | | P04083 | Annexin A1;Annexin | ANXA1 | 1,402,800,000 | 51,347,000 | 99,256,000 | 209,080,000 | 80,360,000 | 733,430,000 | 229,300,000 | 12 |
| Yes | | P02647 | Apolipoprotein A-I;Proapolipoprotein A-I | APOA1 | 1,331,000,000 | 11,399,000 | 117,700,000 | 5,319,000 | 16,475,000 | 1,162,800,000 | 17,217,000 | 15 |
| Yes | | A0A3B3IS80 | Fructose-bisphosphate aldolase B | ALDOB | 1,149,700,000 | 0 | 1,053,900,000 | 0 | 0 | 95,806,000 | 0 | 15 |
| | | P04406 | Glyceraldehyde-3-phosphate dehydrogenase | GAPDH | 1,024,600,000 | 45,228,000 | 381,510,000 | 144,970,000 | 70,343,000 | 219,600,000 | 162,960,000 | 13 |
| | Yes | A0A286YES1 | Ig gamma-3 chain C region | IGHG3 | 1,009,000,000 | 138,810,000 | 318,110,000 | 154,420,000 | 99,280,000 | 160,810,000 | 137,620,000 | 15 |
| | Yes | P02675 | Fibrinogen beta chain | FGB | 987,840,000 | 3,484,400 | 109,660,000 | 9,669,900 | 0 | 843,190,000 | 21,839,000 | 8 |
| | | A0A5F9ZH78 | Arginase-1 | ARG1 | 985,040,000 | 29,882,000 | 296,130,000 | 165,080,000 | 133,750,000 | 191,750,000 | 168,450,000 | 7 |
| | | P04040 | Catalase | CAT | 883,310,000 | 55,685,000 | 377,410,000 | 124,610,000 | 80,278,000 | 132,340,000 | 112,980,000 | 10 |
| | | A0A286YEY1 | Ig alpha-1 chain C region | IGHA1 | 816,250,000 | 19,070,000 | 323,900,000 | 34,146,000 | 95,177,000 | 136,490,000 | 207,470,000 | 11 |
| | Yes | P01023 | Alpha-2-macroglobulin | A2M | 764,980,000 | 22,095,000 | 427,860,000 | 9,797,400 | 18,372,000 | 162,150,000 | 124,700,000 | 19 |
| Yes | | Q2L6G8 | Corneodesmosin | CDSN | 755,530,000 | 85,379,000 | 142,160,000 | 258,560,000 | 81,346,000 | 84,291,000 | 103,800,000 | 5 |
| | Yes | P11021 | 78 kDa glucose-regulated protein | HSPA5 | 754,080,000 | 73,659,000 | 267,450,000 | 111,330,000 | 14,914,000 | 122,090,000 | 164,640,000 | 19 |
| Yes | | Q14103-3 | Heterogeneous nuclear ribonucleoprotein D0 | HNRNPD | 743,760,000 | 190,940,000 | 159,680,000 | 172,750,000 | 62,051,000 | 75,439,000 | 82,904,000 | 7 |
| | Yes | S4R460 | Ig heavy chain V-III region BRO | IGHV3OR16-9 | 737,650,000 | 77,631,000 | 167,330,000 | 68,300,000 | 164,380,000 | 145,600,000 | 114,400,000 | 2 |
| Yes | | Q8N257 | Histone H2B type 3-B | HIST3H2BB | 735,150,000 | 68,866,000 | 97,720,000 | 133,440,000 | 90,551,000 | 222,990,000 | 121,590,000 | 4 |
| | | P02671 | Fibrinogen alpha chain;Fibrinopeptide A | FGA | 727,500,000 | 0 | 39,906,000 | 0 | 0 | 687,600,000 | 0 | 20 |
| Yes | | H0Y9N0 | Alcohol dehydrogenase 4 | ADH4 | 720,950,000 | 0 | 690,910,000 | 0 | 0 | 30,039,000 | 0 | 13 |
| | | A0A6Q8PFJ0 | Prelamin-A/C;Lamin-A/C | LMNA | 692,900,000 | 124,240,000 | 137,300,000 | 102,670,000 | 16,496,000 | 157,450,000 | 154,750,000 | 15 |
| Yes | | P31327 | Carbamoyl-phosphate synthase [ammonia], mitochondrial | CPS1 | 684,130,000 | 0 | 639,470,000 | 0 | 0 | 44,655,000 | 0 | 22 |
| | | P02787 | Serotransferrin | TF | 652,910,000 | 0 | 470,270,000 | 0 | 0 | 146,010,000 | 36,628,000 | 17 |
| | Yes | Q01469 | Fatty acid-binding protein, epidermal | FABP5 | 620,650,000 | 95,889,000 | 59,613,000 | 208,230,000 | 97,652,000 | 68,443,000 | 90,819,000 | 6 |
| Yes | | Q9BTM1 | Histone H2A.J | H2AFJ | 616,220,000 | 134,230,000 | 164,340,000 | 41,711,000 | 94,091,000 | 86,576,000 | 95,268,000 | 3 |
| Yes | | P02042 | Hemoglobin subunit delta | HBD | 608,730,000 | 5,412,700 | 569,100,000 | 0 | 0 | 34,217,000 | 0 | 11 |
| | Yes | P31943 | Heterogeneous nuclear ribonucleoprotein H | HNRNPH1 | 586,320,000 | 235,200,000 | 40,807,000 | 91,509,000 | 52,962,000 | 116,480,000 | 49,360,000 | 10 |
| Yes | | Q15007 | Pre-mRNA-splicing regulator WTAP | WTAP | 583,670,000 | 146,160,000 | 79,564,000 | 83,145,000 | 52,450,000 | 83,328,000 | 139,020,000 | 11 |
| | | E7ETU5 | RNA-binding motif, single-stranded-interacting protein 1 | RBMS1 | 582,990,000 | 101,720,000 | 109,470,000 | 87,943,000 | 47,975,000 | 117,990,000 | 117,880,000 | 4 |
| | | F8VZY9 | Keratin, type I cytoskeletal 18 | KRT18 | 508,470,000 | 182,680,000 | 57,886,000 | 98,079,000 | 48,183,000 | 67,435,000 | 54,210,000 | 11 |
| Yes | | P01024 | Complement C3;Complement C3 beta chain | C3 | 484,020,000 | 0 | 112,750,000 | 0 | 0 | 371,270,000 | 0 | 18 |
| Yes | | F5H5D3 | Tubulin alpha-1C chain;Tubulin alpha-1B chain | TUBA1C | 478,170,000 | 54,611,000 | 141,340,000 | 66,825,000 | 35,734,000 | 118,870,000 | 60,793,000 | 7 |
| Yes | | C9JEU5 | Fibrinogen gamma chain | FGG | 455,740,000 | 0 | 79,865,000 | 2,023,400 | 0 | 368,680,000 | 5,169,500 | 8 |
| Yes | | P31942-2 | Heterogeneous nuclear ribonucleoprotein H3 | HNRNPH3 | 453,350,000 | 48,222,000 | 34,002,000 | 131,920,000 | 77,527,000 | 103,460,000 | 58,216,000 | 7 |
| | Yes | A0A286YEY4 | Ig gamma-2 chain C region | IGHG2 | 432,350,000 | 114,780,000 | 109,100,000 | 83,334,000 | 18,798,000 | 68,437,000 | 37,904,000 | 10 |
| Yes | | P06733 | Alpha-enolase | ENO1 | 410,010,000 | 29,654,000 | 202,800,000 | 42,689,000 | 15,683,000 | 119,190,000 | 0 | 12 |
| | | E9PGY2 | Dynein assembly factor 5, axonemal | DNAAF5 | 404,800,000 | 19,772,000 | 183,310,000 | 67,144,000 | 0 | 69,246,000 | 65,333,000 | 1 |
| Yes | | E5RHP7 | Carbonic anhydrase 1 | CA1 | 401,260,000 | 0 | 391,980,000 | 0 | 0 | 9,284,600 | 0 | 8 |
| | | O43866 | CD5 antigen-like | CD5L | 377,830,000 | 69,239,000 | 55,821,000 | 96,818,000 | 44,043,000 | 63,341,000 | 48,571,000 | 7 |
| | | O14979-3 | Heterogeneous nuclear ribonucleoprotein D-like | HNRNPDL | 365,710,000 | 19,376,000 | 5,685,600 | 198,730,000 | 94,806,000 | 32,417,000 | 14,696,000 | 8 |
| Yes | | Q9UNZ2 | NSFL1 cofactor p47 | NSFL1C | 347,920,000 | 35,893,000 | 47,043,000 | 106,430,000 | 50,500,000 | 26,935,000 | 81,121,000 | 8 |
| | | P01767 | Ig heavy chain V-III region BUT | IGHV3-66 | 341,860,000 | 194,310,000 | 62,905,000 | 0 | 45,120,000 | 39,526,000 | 0 | 3 |
| | | P06702 | Protein S100-A9 | S100A9 | 328,250,000 | 18,939,000 | 0 | 0 | 0 | 309,310,000 | 0 | 5 |
| | | E7EQB2 | Lactotransferrin;Lactoferricin-H | LTF | 327,250,000 | 38,827,000 | 139,660,000 | 27,766,000 | 5,594,100 | 60,142,000 | 55,260,000 | 7 |
| | | J3QSA3 | Ubiquitin-40S ribosomal protein S27a | UBB | 324,440,000 | 21,637,000 | 77,494,000 | 57,113,000 | 32,248,000 | 60,277,000 | 75,668,000 | 2 |
| Yes | | P35579 | Myosin-9 | MYH9 | 312,240,000 | 3,676,700 | 19,015,000 | 0 | 0 | 289,550,000 | 0 | 14 |
| Yes | | P00488 | Coagulation factor XIII A chain | F13A1 | 309,550,000 | 38,304,000 | 144,670,000 | 49,351,000 | 0 | 12,444,000 | 64,783,000 | 11 |
| | | P16403 | Histone H1.2 | HIST1H1C | 308,980,000 | 26,941,000 | 14,080,000 | 6,953,300 | 0 | 29,397,000 | 231,610,000 | 8 |
| Yes | | P05091 | Aldehyde dehydrogenase, mitochondrial | ALDH2 | 295,420,000 | 0 | 295,420,000 | 0 | 0 | 0 | 0 | 13 |
| | Yes | P31944 | Caspase-14;Caspase-14 subunit p17, mature form | CASP14 | 292,460,000 | 41,450,000 | 20,154,000 | 92,287,000 | 47,890,000 | 24,225,000 | 66,452,000 | 5 |
| Yes | | P23141-3 | Liver carboxylesterase 1 | CES1 | 287,760,000 | 0 | 275,240,000 | 0 | 0 | 12,513,000 | 0 | 8 |
| | | Q13492-3 | Phosphatidylinositol-binding clathrin assembly protein | PICALM | 280,250,000 | 0 | 13,085,000 | 54,721,000 | 40,016,000 | 68,106,000 | 104,330,000 | 2 |
| Yes | | P08263 | Glutathione S-transferase A1 | GSTA1 | 276,080,000 | 0 | 276,080,000 | 0 | 0 | 0 | 0 | 6 |
| | | P01709 | Ig lambda chain V-II region MGC | | 272,320,000 | 194,040,000 | 27,482,000 | 0 | 0 | 20,496,000 | 30,296,000 | 1 |
| Yes | | K7EMS3 | Keratin, type I cytoskeletal 19 | KRT19 | 271,550,000 | 43,143,000 | 35,179,000 | 52,355,000 | 28,751,000 | 3,693,300 | 108,430,000 | 8 |
| Yes | | P32119 | Peroxiredoxin-2 | PRDX2 | 264,970,000 | 10,440,000 | 193,050,000 | 17,114,000 | 0 | 28,665,000 | 15,708,000 | 7 |
| | | P11142 | Heat shock cognate 71 kDa protein | HSPA8 | 256,440,000 | 21,135,000 | 64,536,000 | 39,021,000 | 0 | 87,034,000 | 44,712,000 | 12 |
| Yes | | H3BQ34 | Pyruvate kinase;Pyruvate kinase PKM | PKM | 253,600,000 | 157,610,000 | 12,316,000 | 26,633,000 | 7,716,200 | 41,154,000 | 8,175,000 | 4 |
| Yes | | M0QZK8 | Gamma-glutamylcyclotransferase | GGCT | 252,790,000 | 23,067,000 | 23,773,000 | 53,047,000 | 57,290,000 | 26,710,000 | 68,899,000 | 3 |
| | | P62805 | Histone H4 | HIST1H4A | 247,550,000 | 32,165,000 | 44,025,000 | 57,166,000 | 32,274,000 | 24,152,000 | 57,770,000 | 3 |
| | | F8VV32 | Lysozyme;Lysozyme C | LYZ | 237,840,000 | 25,942,000 | 54,297,000 | 24,124,000 | 46,944,000 | 38,336,000 | 48,197,000 | 3 |
| Yes | | A0A2R8YGD1 | Tripeptidyl-peptidase 1 | TPP1 | 228,720,000 | 59,580,000 | 36,930,000 | 40,003,000 | 12,722,000 | 48,382,000 | 31,107,000 | 2 |
| | | A0A286YFJ8 | Ig gamma-4 chain C region | IGHG4 | 221,300,000 | 21,901,000 | 25,795,000 | 0 | 48,084,000 | 94,876,000 | 30,645,000 | 9 |
| Yes | | P30086 | Phosphatidylethanolamine-binding protein 1 | PEBP1 | 220,410,000 | 0 | 220,410,000 | 0 | 0 | 0 | 0 | 6 |
| | | Q8NDC0 | MAPK-interacting and spindle-stabilizing protein-like | MAPK1IP1L | 219,790,000 | 0 | 0 | 74,677,000 | 0 | 55,083,000 | 90,029,000 | 1 |
| Yes | | E9PSB3 | Eukaryotic translation initiation factor 3 Sub M | EIF3M | 204,390,000 | 0 | 0 | 204,390,000 | 0 | 0 | 0 | 1 |
| Yes | | P00367 | Glutamate dehydrogenase 1, mitochondrial | GLUD1 | 202,320,000 | 0 | 194,820,000 | 0 | 0 | 7,505,800 | 0 | 8 |
| Yes | | P01040 | Cystatin-A;Cystatin-A, N-terminally processed | CSTA | 200,650,000 | 31,866,000 | 28,575,000 | 24,874,000 | 17,883,000 | 0 | 97,455,000 | 3 |
| Yes | | H0Y755 | Low affinity immunoglobulin gamma Fc region receptor III-A | FCGR3A | 200,010,000 | 96,729,000 | 0 | 76,577,000 | 15,235,000 | 0 | 11,468,000 | 2 |
| Yes | | Q6UWP8 | Suprabasin | SBSN | 199,090,000 | 23,686,000 | 5,567,100 | 53,784,000 | 11,163,000 | 9,392,600 | 95,502,000 | 7 |
| Yes | | H0YH81 | ATP synthase subunit beta,mitochondrial | ATP5B | 192,440,000 | 0 | 150,240,000 | 11,186,000 | 0 | 26,174,000 | 4,835,000 | 3 |
| Yes | | P00738 | Haptoglobin;Haptoglobin alpha chain | HP | 191,600,000 | 0 | 83,217,000 | 0 | 0 | 105,830,000 | 2,547,800 | 8 |
| | Yes | P05109 | Protein S100-A8;Protein S100-A8, N-terminally processed | S100A8 | 188,970,000 | 15,112,000 | 51,461,000 | 6,955,600 | 7,752,500 | 102,410,000 | 5,286,800 | 4 |
| | | P10412 | Histone H1.4 | HIST1H1E | 188,770,000 | 0 | 51,503,000 | 19,340,000 | 0 | 38,303,000 | 79,622,000 | 7 |
| | | P30101 | Protein disulfide-isomerase A3 | PDIA3 | 184,850,000 | 27,987,000 | 81,404,000 | 9,267,600 | 0 | 52,967,000 | 13,224,000 | 7 |
| Yes | | Q60FE5 | Filamin-A | FLNA | 176,900,000 | 17,314,000 | 62,222,000 | 4,156,600 | 0 | 93,211,000 | 0 | 14 |
| | Yes | P59666 | Neutrophil defensin 3;HP 3-56;Neutrophil defensin 2 | DEFA3 | 175,940,000 | 12,525,000 | 147,180,000 | 0 | 0 | 0 | 16,235,000 | 4 |
| | | A0A7I2V4I6 | Heterogeneous nuclear ribonucleoproteins A2/B1 | HNRNPA2B1 | 175,770,000 | 10,611,000 | 33,709,000 | 28,025,000 | 3,763,900 | 47,208,000 | 52,448,000 | 8 |
| | | C9JZN1 | Guanine nucleotide-binding protein G(I)/G(S)/G(T) subunit 2 | GNB2 | 167,230,000 | 0 | 32,409,000 | 0 | 114,540,000 | 0 | 20,285,000 | 3 |
| Yes | | E9PJX3 | DC-STAMP domain-containing protein 1 | DCST1 | 165,830,000 | 165,830,000 | 0 | 0 | 0 | 0 | 0 | 1 |
| Yes | | Q93088 | Betaine--homocysteine S-methyltransferase 1 | BHMT | 156,930,000 | 0 | 156,930,000 | 0 | 0 | 0 | 0 | 5 |
| Yes | | Q08188 | Protein-glutamine gamma-glutamyltransferase E | TGM3 | 151,040,000 | 19,454,000 | 14,749,000 | 30,930,000 | 27,901,000 | 11,456,000 | 46,554,000 | 6 |
| | | Q5JP53 | Tubulin beta chain;Tubulin beta-2B chain | TUBB | 148,810,000 | 0 | 39,759,000 | 10,457,000 | 5,418,600 | 93,172,000 | 0 | 3 |
| Yes | | P00352 | Retinal dehydrogenase 1 | ALDH1A1 | 147,820,000 | 0 | 143,910,000 | 0 | 0 | 3,905,500 | 0 | 7 |
| | | A0A7I2V3H3 | Putative elongation factor 1-alpha-like 3 | EEF1A1 | 146,880,000 | 10,829,000 | 56,560,000 | 10,460,000 | 7,325,300 | 52,315,000 | 9,388,600 | 1 |
| Yes | | P29508-2 | Serpin B3 | SERPINB3 | 144,360,000 | 34,074,000 | 0 | 63,249,000 | 7,558,700 | 7,745,700 | 31,729,000 | 3 |
| | | P47929 | Galectin-7 | LGALS7 | 142,190,000 | 14,374,000 | 0 | 53,808,000 | 19,822,000 | 41,851,000 | 12,331,000 | 4 |
| Yes | | P02730 | Band 3 anion transport protein | SLC4A1 | 137,560,000 | 0 | 122,820,000 | 6,434,500 | 0 | 0 | 8,304,400 | 6 |
| Yes | | P25705-2 | ATP synthase subunit alpha, mitochondrial | ATP5A1 | 135,600,000 | 25,045,000 | 92,411,000 | 4,146,300 | 0 | 14,002,000 | 0 | 9 |
| Yes | | P09467 | Fructose-1,6-bisphosphatase 1 | FBP1 | 134,090,000 | 0 | 112,310,000 | 0 | 0 | 21,781,000 | 0 | 4 |
| Yes | | A0A6Q8PFK8 | Heat shock protein beta-1 | HSPB1 | 129,770,000 | 6,293,600 | 23,017,000 | 23,662,000 | 14,009,000 | 27,850,000 | 34,937,000 | 3 |
| | | P14625 | Endoplasmin | HSP90B1 | 128,220,000 | 0 | 87,804,000 | 12,003,000 | 1,880,000 | 22,276,000 | 4,253,300 | 6 |
| | | A0A5F9ZHX5 | Chromodomain-helicase-DNA-binding protein 3 | CHD3 | 126,410,000 | 72,151,000 | 0 | 0 | 0 | 0 | 54,262,000 | 1 |
| | | D3YTK1 | B-cell lymphoma/leukemia 11B | BCL11B | 124,710,000 | 76,098,000 | 14,068,000 | 16,701,000 | 0 | 0 | 17,845,000 | 1 |
| Yes | | P07476 | Involucrin | IVL | 120,360,000 | 0 | 0 | 0 | 0 | 120,360,000 | 0 | 6 |
| Yes | | P00558-2 | Phosphoglycerate kinase 1 | PGK1 | 119,430,000 | 0 | 106,620,000 | 0 | 0 | 12,816,000 | 0 | 5 |
| | | A0A0G2JRN3 | Alpha-1-antitrypsin;Short peptide from AAT | SERPINA1 | 119,420,000 | 0 | 0 | 0 | 0 | 119,420,000 | 0 | 2 |
| Yes | | P10599-2 | Thioredoxin | TXN | 119,320,000 | 32,992,000 | 7,421,500 | 15,014,000 | 6,942,900 | 38,357,000 | 18,590,000 | 3 |
| | | A0A087WVQ6 | Clathrin heavy chain;Clathrin heavy chain 1 | CLTC | 118,860,000 | 7,694,400 | 39,319,000 | 4,351,100 | 5,094,400 | 27,799,000 | 34,607,000 | 5 |

| | | | | | | | | | | | | |
|---|---|---|---|---|---|---|---|---|---|---|---|---|
| | | P11166 | Solute carrier family 2, facilitated glucose transporter member 1 | SLC2A1 | 117,840,000 | 27,594,000 | 20,800,000 | 27,638,000 | 17,045,000 | 17,769,000 | 6,991,000 | 1 |
| Yes | | A0A0A0MSI0 | Peroxiredoxin-1 | PRDX1 | 117,410,000 | 0 | 23,434,000 | 24,158,000 | 0 | 50,913,000 | 18,908,000 | 6 |
| | | P04843 | Dolichyl-diphosphooligosaccharide--protein glycosyltransferase subunit 1 | RPN1 | 114,750,000 | 14,069,000 | 19,291,000 | 38,305,000 | 0 | 5,918,100 | 37,166,000 | 5 |
| | | Q9BS26 | Endoplasmic reticulum resident protein 44 | ERP44 | 114,410,000 | 0 | 19,792,000 | 0 | 57,777,000 | 15,611,000 | 21,226,000 | 2 |
| Yes | | P31040 | Succinate dehydrogenase [ubiquinone] flavoprotein subunit, mitochondrial | SDHA | 110,870,000 | 21,023,000 | 29,762,000 | 5,004,900 | 6,511,700 | 7,760,200 | 40,805,000 | 5 |
| | | E9PS23 | Cofilin-1 | CFL1 | 105,680,000 | 0 | 47,412,000 | 6,922,800 | 6,769,200 | 42,374,000 | 2,206,100 | 2 |
| Yes | | P00918 | Carbonic anhydrase 2 | CA2 | 103,530,000 | 0 | 103,530,000 | 0 | 0 | 0 | 0 | 6 |
| Yes | | P40926 | Malate dehydrogenase, mitochondrial;Malate dehydrogenase | MDH2 | 102,110,000 | 0 | 102,110,000 | 0 | 0 | 0 | 0 | 6 |
| Yes | | P09210 | Glutathione S-transferase A2 | GSTA2 | 101,640,000 | 0 | 101,640,000 | 0 | 0 | 0 | 0 | 6 |
| Yes | | P09211 | Glutathione S-transferase P | GSTP1 | 101,360,000 | 0 | 24,528,000 | 0 | 0 | 76,833,000 | 0 | 2 |
| Yes | | P00338-3 | L-lactate dehydrogenase A chain | LDHA | 101,020,000 | 0 | 67,486,000 | 0 | 0 | 33,536,000 | 0 | 4 |
| Yes | | P30084 | Enoyl-CoA hydratase, mitochondrial | ECHS1 | 100,510,000 | 0 | 100,510,000 | 0 | 0 | 0 | 0 | 5 |
| Yes | | P00326 | Alcohol dehydrogenase 1C | ADH1C | 99,362,000 | 0 | 99,362,000 | 0 | 0 | 0 | 0 | 12 |
| Yes | | P11182 | Lipoamide acyltransferase component of branched-chain α-keto acid | DBT | 98,020,000 | 2,429,500 | 0 | 88,425,000 | 7,165,200 | 0 | 0 | 6 |
| Yes | | P60174 | Triosephosphate isomerase | TPI1 | 96,410,000 | 0 | 96,410,000 | 0 | 0 | 0 | 0 | 6 |
| Yes | | P31995-4 | Low affinity immunoglobulin gamma Fc region receptor II-c | FCGR2C | 95,150,000 | 0 | 0 | 21,613,000 | 16,029,000 | 0 | 57,508,000 | 2 |
| Yes | | P81605-2 | Dermcidin;Survival-promoting peptide;DCD-1 | DCD | 94,948,000 | 0 | 17,310,000 | 24,755,000 | 14,705,000 | 27,485,000 | 10,693,000 | 8 |
| | | A0A7I2V2R3 | Heterogeneous nuclear ribonucleoprotein A3 | HNRNPA3 | 94,476,000 | 0 | 0 | 40,480,000 | 18,442,000 | 11,950,000 | 23,604,000 | 5 |
| Yes | | Q15828 | Cystatin-M | CST6 | 90,006,000 | 23,842,000 | 0 | 12,469,000 | 11,616,000 | 14,124,000 | 27,955,000 | 1 |
| Yes | | P04259 | Keratin, type II cytoskeletal 6B | KRT6B | 89,853,000 | 0 | 3,936,600 | 12,262,000 | 4,134,500 | 69,520,000 | 0 | 56 |
| | | K7EQ02 | DAZ-associated protein 1 | DAZAP1 | 88,043,000 | 0 | 4,283,400 | 36,199,000 | 21,232,000 | 26,328,000 | 0 | 2 |
| Yes | | P08238 | Heat shock protein HSP 90-beta | HSP90AB1 | 87,486,000 | 9,004,500 | 43,177,000 | 0 | 0 | 26,031,000 | 9,274,000 | 8 |
| Yes | | Q13835-2 | Plakophilin-1 | PKP1 | 87,271,000 | 14,367,000 | 4,843,200 | 0 | 14,614,000 | 47,376,000 | 6,070,300 | 3 |
| | | P16401 | Histone H1.5 | HIST1H1B | 86,334,000 | 0 | 0 | 0 | 0 | 33,838,000 | 52,496,000 | 5 |
| Yes | | P21549 | Serine--pyruvate aminotransferase | AGXT | 84,253,000 | 0 | 84,253,000 | 0 | 0 | 0 | 0 | 3 |
| Yes | | E7EQR4 | Ezrin | EZR | 83,177,000 | 11,616,000 | 57,174,000 | 0 | 0 | 0 | 14,386,000 | 6 |
| Yes | | A8MW49 | Fatty acid-binding protein, liver | FABP1 | 80,281,000 | 0 | 80,281,000 | 0 | 0 | 0 | 0 | 3 |
| Yes | | Q09666 | Neuroblast differentiation-associated protein AHNAK | AHNAK | 79,609,000 | 4,552,900 | 0 | 0 | 0 | 75,056,000 | 0 | 8 |
| Yes | | P06396-2 | Gelsolin | GSN | 77,391,000 | 0 | 0 | 0 | 0 | 77,391,000 | 0 | 5 |
| Yes | | P08311 | Cathepsin G | CTSG | 75,345,000 | 0 | 64,752,000 | 0 | 0 | 10,593,000 | 0 | 3 |
| Yes | | P07737 | Profilin-1 | PFN1 | 73,460,000 | 0 | 45,606,000 | 0 | 0 | 27,854,000 | 0 | 2 |
| Yes | | Q13867 | Bleomycin hydrolase | BLMH | 71,299,000 | 15,720,000 | 8,186,000 | 22,679,000 | 0 | 9,475,800 | 15,237,000 | 2 |
| Yes | | A0A0G2JIW1 | Heat shock 70 kDa protein 1A;Heat shock 70 kDa protein 1B | HSPA1A | 70,838,000 | 0 | 40,124,000 | 9,828,700 | 0 | 14,307,000 | 6,577,900 | 7 |
| Yes | | P25311 | Zinc-alpha-2-glycoprotein | AZGP1 | 70,437,000 | 17,679,000 | 10,057,000 | 36,466,000 | 0 | 0 | 6,235,100 | 6 |
| | | A0A7I2YQK6 | 60 kDa heat shock protein, mitochondrial | HSPD1 | 70,230,000 | 0 | 41,023,000 | 0 | 0 | 29,206,000 | 0 | 3 |
| Yes | | P30041 | Peroxiredoxin-6 | PRDX6 | 70,214,000 | 0 | 70,214,000 | 0 | 0 | 0 | 0 | 2 |
| Yes | | P17066 | Heat shock 70 kDa protein 6;Putative heat shock 70 kDa protein 7 | HSPA6 | 69,823,000 | 0 | 31,296,000 | 5,901,700 | 4,534,000 | 18,897,000 | 9,194,300 | 4 |
| | Yes | E9PHT9 | Annexin;Annexin A5 | ANXA5 | 69,705,000 | 7,186,600 | 4,930,200 | 11,971,000 | 16,361,000 | 5,489,900 | 23,766,000 | 4 |
| | | P31025 | Lipocalin-1;Putative lipocalin 1-like protein 1 | LCN1 | 68,579,000 | 17,246,000 | 0 | 8,715,500 | 19,543,000 | 9,898,400 | 13,176,000 | 2 |
| | | P31930 | Cytochrome b-c1 complex subunit 1, mitochondrial | UQCRC1 | 66,759,000 | 0 | 7,055,500 | 32,142,000 | 9,727,500 | 17,834,000 | 0 | 2 |
| Yes | | P22735 | Protein-glutamine gamma-glutamyltransferase K | TGM1 | 66,068,000 | 9,145,000 | 8,097,100 | 11,141,000 | 12,123,000 | 8,823,300 | 16,739,000 | 2 |
| Yes | | Q6ZVX7 | F-box only protein 50 | NCCRP1 | 65,548,000 | 18,299,000 | 1,627,800 | 13,871,000 | 10,069,000 | 9,179,600 | 12,502,000 | 2 |
| Yes | | Q92637 | High affinity immunoglobulin gamma Fc receptor IB | FCGR1B | 65,085,000 | 14,808,000 | 24,911,000 | 0 | 0 | 0 | 25,367,000 | 2 |
| | | P31947-2 | 14-3-3 protein sigma | SFN | 65,014,000 | 0 | 0 | 0 | 0 | 65,014,000 | 0 | 4 |
| | | M0R3F1 | Heterogeneous nuclear ribonucleoprotein U-like protein 1 | HNRNPUL1 | 64,921,000 | 7,997,300 | 41,310,000 | 15,614,000 | 0 | 0 | 0 | 3 |
| | | Q9HAU0-6 | Pleckstrin homology domain-6 | PLEKHA6 | 64,712,000 | 0 | 0 | 0 | 35,710,000 | 13,996,000 | 15,006,000 | 1 |
| Yes | | Q16822 | Phosphoenolpyruvate carboxykinase [GTP], mitochondrial | PCK2 | 63,356,000 | 0 | 63,356,000 | 0 | 0 | 0 | 0 | 6 |
| Yes | | P42765 | 3-ketoacyl-CoA thiolase, mitochondrial | ACAA2 | 63,130,000 | 0 | 63,130,000 | 0 | 0 | 0 | 0 | 3 |
| Yes | | V9GYC1 | Apolipoprotein A-II;Proapolipoprotein A-II;Truncated apolipoprotein A-II | APOA2 | 63,025,000 | 0 | 0 | 0 | 0 | 63,025,000 | 0 | 3 |
| Yes | | O95954 | Formimidoyltransferase-cyclodeaminase | FTCD | 62,955,000 | 0 | 62,955,000 | 0 | 0 | 0 | 0 | 3 |
| | | P01833 | Polymeric immunoglobulin receptor;Secretory component | PIGR | 62,780,000 | 55,565,000 | 0 | 7,215,700 | 0 | 0 | 0 | 2 |
| Yes | | H3BUH7 | Fructose-bisphosphate aldolase;Fructose-bisphosphate aldolase A | ALDOA | 61,547,000 | 0 | 30,590,000 | 0 | 0 | 30,957,000 | 0 | 2 |
| Yes | | I3NI03 | Protein disulfide-isomerase | P4HB | 61,057,000 | 5,594,500 | 24,393,000 | 6,481,000 | 4,107,900 | 14,479,000 | 6,001,800 | 1 |
| Yes | | P04899-4 | Guanine nucleotide-binding protein G(i) subunit alpha-2 | GNAI2 | 60,628,000 | 0 | 5,045,200 | 17,245,000 | 6,652,900 | 12,318,000 | 19,367,000 | 3 |
| Yes | | J3QS36 | L-xylulose reductase | DCXR | 58,887,000 | 0 | 58,887,000 | 0 | 0 | 0 | 0 | 4 |
| Yes | | D6REM4 | Casein kinase I isoform alpha;Casein kinase I isoform alpha-like | CSNK1A1 | 58,574,000 | 58,574,000 | 0 | 0 | 0 | 0 | 0 | 1 |
| | | P01594 | Ig kappa chain V-I region AU;Ig kappa chain V-I region AG | IGKV1D-33 | 58,512,000 | 35,985,000 | 4,295,800 | 2,809,900 | 3,517,000 | 4,290,800 | 7,612,700 | 1 |
| | | A0A0A0MSV6 | Complement C1q subcomponent subunit B | C1QB | 58,010,000 | 0 | 0 | 58,010,000 | 0 | 0 | 0 | 2 |
| Yes | | P69891 | Hemoglobin subunit gamma-1 | HBG1 | 57,351,000 | 0 | 0 | 20,525,000 | 11,885,000 | 0 | 24,940,000 | 11 |
| Yes | | Q5T6W2 | Heterogeneous nuclear ribonucleoprotein K | HNRNPK | 56,797,000 | 0 | 9,694,800 | 10,343,000 | 0 | 20,754,000 | 16,006,000 | 1 |
| Yes | | Q6P0N6 | Dystonin | DST | 55,054,000 | 0 | 0 | 0 | 55,054,000 | 0 | 0 | 1 |
| Yes | | P08559-3 | Pyruvate dehydrogenase E1 component subunit alpha, mitochondrial | PDHA1 | 54,649,000 | 0 | 0 | 36,915,000 | 17,733,000 | 0 | 0 | 2 |
| Yes | | P54868 | Hydroxymethylglutaryl-CoA synthase, mitochondrial | HMGCS2 | 54,294,000 | 0 | 54,294,000 | 0 | 0 | 0 | 0 | 3 |
| Yes | | K7EP41 | Glucose-6-phosphate isomerase | GPI | 51,787,000 | 0 | 28,093,000 | 0 | 0 | 23,693,000 | 0 | 1 |
| | | A0A7I2V5R8 | Dipeptidyl peptidase 4 soluble form | DPP4 | 49,250,000 | 16,947,000 | 0 | 6,971,900 | 0 | 0 | 25,332,000 | 3 |
| Yes | | P02745 | Complement C1q subcomponent subunit A | C1QA | 49,029,000 | 0 | 0 | 26,241,000 | 7,260,800 | 0 | 15,527,000 | 1 |
| Yes | | H7C131 | 3-ketoacyl-CoA thiolase, peroxisomal | ACAA1 | 49,012,000 | 0 | 39,040,000 | 0 | 0 | 9,971,800 | 0 | 2 |
| Yes | | P14550 | Alcohol dehydrogenase [NADP(+)] | AKR1A1 | 48,970,000 | 0 | 48,970,000 | 0 | 0 | 0 | 0 | 3 |
| Yes | | H7BYH4 | Superoxide dismutase [Cu-Zn] | SOD1 | 48,641,000 | 0 | 36,536,000 | 0 | 0 | 12,105,000 | 0 | 1 |
| Yes | | O75891-2 | Cytosolic 10-formyltetrahydrofolate dehydrogenase | ALDH1L1 | 47,334,000 | 0 | 47,334,000 | 0 | 0 | 0 | 0 | 4 |
| Yes | | P16152 | Carbonyl reductase [NADPH] 1 | CBR1 | 46,537,000 | 0 | 46,537,000 | 0 | 0 | 0 | 0 | 3 |
| Yes | | Q16762 | Thiosulfate sulfurtransferase | TST | 46,314,000 | 0 | 46,314,000 | 0 | 0 | 0 | 0 | 3 |
| | | P98082-2 | Disabled homolog 2 | DAB2 | 46,020,000 | 7,661,800 | 4,233,100 | 4,780,500 | 11,910,000 | 4,537,100 | 12,898,000 | 1 |
| Yes | | A0A2R8Y7L2 | Peroxisomal multifunctional enzyme type 2;Enoyl-CoA hydratase 2 | HSD17B4 | 44,462,000 | 0 | 44,462,000 | 0 | 0 | 0 | 0 | 3 |
| Yes | | F5H5P2 | 2-oxoisovalerate dehydrogenase subunit alpha, mitochondrial | BCKDHA | 44,336,000 | 0 | 0 | 27,748,000 | 6,992,100 | 9,596,700 | 0 | 2 |
| | | P07910 | Heterogeneous nuclear ribonucleoproteins C1/C2 | HNRNPC | 44,199,000 | 0 | 0 | 19,282,000 | 0 | 24,917,000 | 0 | 2 |
| | | P01701 | Ig lambda chain V-I region NEW | IGLV1-51 | 43,260,000 | 43,260,000 | 0 | 0 | 0 | 0 | 0 | 1 |
| Yes | | Q16610-2 | Extracellular matrix protein 1 | ECM1 | 43,164,000 | 16,073,000 | 0 | 4,679,200 | 16,017,000 | 0 | 6,394,400 | 3 |
| Yes | | P80748 | Ig lambda chain V-III region LOI | IGLV3-9 | 39,938,000 | 39,938,000 | 0 | 0 | 0 | 0 | 0 | 2 |
| Yes | | P00480 | Ornithine carbamoyltransferase, mitochondrial | OTC | 38,811,000 | 0 | 38,811,000 | 0 | 0 | 0 | 0 | 3 |
| Yes | | P06727 | Apolipoprotein A-IV | APOA4 | 38,730,000 | 0 | 0 | 0 | 0 | 38,730,000 | 0 | 4 |
| | Yes | A0A3B3ISA6 | Complement C4-A;Complement C4 beta chain | C4B | 37,477,000 | 0 | 0 | 0 | 0 | 37,477,000 | 0 | 3 |
| | | C9J2I0 | Arf-GAP domain and FG repeat-containing protein 1 | AGFG1 | 37,463,000 | 0 | 19,993,000 | 0 | 17,470,000 | 0 | 0 | 1 |
| Yes | | P62081 | 40S ribosomal protein S7 | RPS7 | 36,563,000 | 0 | 0 | 0 | 0 | 36,563,000 | 0 | 1 |
| Yes | | P00505 | Aspartate aminotransferase, mitochondrial | GOT2 | 36,173,000 | 0 | 36,173,000 | 0 | 0 | 0 | 0 | 1 |
| Yes | | Q14669 | E3 ubiquitin-protein ligase TRIP12 | TRIP12 | 35,387,000 | 5,063,600 | 0 | 0 | 1,274,500 | 7,478,400 | 21,571,000 | 2 |
| Yes | | P00747 | Plasminogen;Plasmin heavy chain A;Activation peptide;Angiostatin | PLG | 35,204,000 | 0 | 18,546,000 | 0 | 0 | 16,658,000 | 0 | 2 |
| Yes | | A0A5F9ZHD4 | Acetyl-CoA acetyltransferase, mitochondrial | ACAT1 | 34,864,000 | 0 | 34,864,000 | 0 | 0 | 0 | 0 | 1 |
| Yes | | P42357 | Histidine ammonia-lyase | HAL | 34,798,000 | 3,023,300 | 5,789,400 | 4,315,200 | 4,655,100 | 3,693,900 | 13,321,000 | 4 |
| Yes | | P08574 | Cytochrome c1, heme protein, mitochondrial | CYC1 | 34,705,000 | 17,970,000 | 0 | 0 | 3,321,100 | 6,646,500 | 6,767,300 | 1 |
| Yes | | A0A7I2V2G2 | Stress-70 protein, mitochondrial | HSPA9 | 34,422,000 | 3,644,700 | 30,777,000 | 0 | 0 | 0 | 0 | 5 |
| | | Q13151 | Heterogeneous nuclear ribonucleoprotein A0 | HNRNPA0 | 34,332,000 | 9,337,700 | 8,802,000 | 6,967,600 | 9,225,100 | 0 | 0 | 1 |
| Yes | | Q00266 | S-adenosylmethionine synthase isoform type-1 | MAT1A | 34,266,000 | 0 | 34,266,000 | 0 | 0 | 0 | 0 | 3 |
| | | B4E1S2 | Annexin;Annexin A4 | ANXA4 | 34,107,000 | 0 | 23,248,000 | 7,248,900 | 0 | 0 | 3,610,400 | 2 |
| Yes | | E7EN95 | Filamin-B | FLNB | 33,248,000 | 0 | 0 | 0 | 0 | 33,248,000 | 0 | 4 |
| Yes | | Q15149-7 | Plectin | PLEC | 32,622,000 | 0 | 0 | 0 | 0 | 32,622,000 | 0 | 5 |
| Yes | | Q5T750 | Skin-specific protein 32 | XP32 | 32,441,000 | 14,229,000 | 12,308,000 | 0 | 5,905,100 | 0 | 0 | 1 |
| | | P29401 | Transketolase | TKT | 32,236,000 | 0 | 25,916,000 | 0 | 0 | 6,320,100 | 0 | 4 |
| Yes | | R4GN49 | Protein S100-A2 | S100A2 | 32,039,000 | 0 | 0 | 0 | 0 | 32,039,000 | 0 | 2 |
| Yes | | Q14574-2 | Desmocollin-3 | DSC3 | 31,820,000 | 0 | 0 | 11,287,000 | 9,170,400 | 0 | 11,362,000 | 2 |
| Yes | | P07900 | Heat shock protein HSP 90-alpha | HSP90AA1 | 31,748,000 | 0 | 0 | 18,987,000 | 0 | 12,762,000 | 0 | 7 |
| Yes | | K7ESE1 | Keratin, type I cytoskeletal 17 | KRT17 | 31,444,000 | 0 | 0 | 0 | 0 | 31,444,000 | 0 | 6 |
| | | H7BZJ3 | Protein disulfide-isomerase A3 | PDIA3 | 31,218,000 | 10,969,000 | 0 | 0 | 0 | 12,027,000 | 8,221,900 | 2 |
| Yes | | A0A2R8YGX3 | Tropomyosin alpha-4 chain | TPM4 | 30,416,000 | 0 | 0 | 0 | 0 | 30,416,000 | 0 | 4 |
| | | A0A1X7SBZ2 | Probable ATP-dependent RNA helicase DDX17 | DDX17 | 30,189,000 | 11,040,000 | 12,105,000 | 0 | 0 | 7,044,500 | 0 | 1 |
| Yes | | P68133 | Actin, alpha skeletal muscle; Actin, aortic smooth muscle | ACTA1 | 28,542,000 | 0 | 0 | 22,625,000 | 0 | 5,916,600 | 0 | 11 |
| Yes | | P31151 | Protein S100-A7 | S100A7 | 28,377,000 | 16,068,000 | 0 | 0 | 3,923,400 | 5,362,900 | 3,022,700 | 1 |
| Yes | | O75874 | Isocitrate dehydrogenase [NADP] cytoplasmic | IDH1 | 27,456,000 | 0 | 27,456,000 | 0 | 0 | 0 | 0 | 3 |
| Yes | | P63104 | 14-3-3 protein zeta/delta | YWHAZ | 26,529,000 | 0 | 0 | 3,983,100 | 0 | 11,736,000 | 10,810,000 | 3 |
| Yes | | A0A7I2V3U0 | Aconitate hydratase, mitochondrial | ACO2 | 26,341,000 | 0 | 7,668,600 | 3,421,200 | 0 | 7,748,000 | 7,503,500 | 2 |
| Yes | | C9JEH7 | 40S ribosomal protein S4, X isoform | RPS4Y1 | 26,072,000 | 26,072,000 | 0 | 0 | 0 | 0 | 0 | 3 |
| Yes | | D6RHJ7 | Dihydropteridine reductase | QDPR | 25,964,000 | 0 | 22,627,000 | 0 | 0 | 3,337,100 | 0 | 2 |
| Yes | | P21980 | Protein-glutamine gamma-glutamyltransferase 2 | TGM2 | 25,527,000 | 0 | 25,527,000 | 0 | 0 | 0 | 0 | 3 |
| | Yes | I3L1P8 | Mitochondrial 2-oxoglutarate/malate carrier protein | SLC25A11 | 25,396,000 | 0 | 0 | 9,416,800 | 0 | 5,550,200 | 10,429,000 | 1 |
| Yes | | E9PP21 | Cysteine and glycine-rich protein 1 | CSRP1 | 25,181,000 | 0 | 19,963,000 | 5,218,000 | 0 | 0 | 0 | 1 |
| Yes | | P26641 | Elongation factor 1-gamma | EEF1G | 25,062,000 | 8,120,000 | 0 | 0 | 0 | 16,942,000 | 0 | 2 |
| Yes | | Q99497 | Protein deglycase DJ-1 | PARK7 | 24,469,000 | 0 | 24,469,000 | 0 | 0 | 0 | 0 | 2 |
| | | B4DK69 | Aldo-keto reductase family 1 member C2 | AKR1C2 | 23,775,000 | 0 | 23,775,000 | 0 | 0 | 0 | 0 | 3 |
| Yes | | H3BTX9 | Acyl-coenzyme A synthetase ACSM2B, mitochondrial | ACSM2B | 23,403,000 | 0 | 23,403,000 | 0 | 0 | 0 | 0 | 2 |
| | | P17096 | High mobility group protein HMG-I/HMG-Y | HMGA1 | 23,239,000 | 0 | 0 | 0 | 0 | 0 | 23,239,000 | 1 |
| Yes | | O43548 | Protein-glutamine gamma-glutamyltransferase 5 | TGM5 | 22,931,000 | 0 | 0 | 5,106,200 | 3,459,400 | 5,197,500 | 9,168,100 | 1 |
| Yes | | A0A6E1W9L1 | Zinc finger and BTB domain-containing protein 3 | ZBTB3 | 22,345,000 | 5,533,200 | 0 | 7,473,000 | 0 | 9,338,700 | 0 | 2 |
| Yes | | P07099 | Epoxide hydrolase 1 | EPHX1 | 21,730,000 | 0 | 21,730,000 | 0 | 0 | 0 | 0 | 2 |
| Yes | | A0A7I2YQ74 | UTP--glucose-1-phosphate uridylyltransferase | UGP2 | 21,651,000 | 0 | 21,651,000 | 0 | 0 | 0 | 0 | 3 |
| Yes | | P34896-3 | Serine hydroxymethyltransferase, cytosolic | SHMT1 | 21,544,000 | 0 | 21,544,000 | 0 | 0 | 0 | 0 | 2 |
| Yes | | P51857-2 | 3-oxo-5-beta-steroid 4-dehydrogenase | AKR1D1 | 21,175,000 | 0 | 21,175,000 | 0 | 0 | 0 | 0 | 2 |
| Yes | | H0YCY6 | Bifunctional ATP-dependent dihydroxyacetone kinase; ATP-dependent | TKFC | 20,829,000 | 0 | 20,829,000 | 0 | 0 | 0 | 0 | 3 |
| Yes | | F8VPF3 | Myosin light polypeptide 6;Myosin light chain 6B | MYL6 | 20,561,000 | 0 | 0 | 0 | 0 | 20,561,000 | 0 | 1 |
| Yes | | P01019 | Angiotensinogen;Angiotensin-1;Angiotensin-2;Angiotensin-3;Angiotensin-4 | AGT | 20,374,000 | 0 | 0 | 0 | 0 | 20,374,000 | 0 | 2 |

| | | | | | | | | | | | | |
|---|---|---|---|---|---|---|---|---|---|---|---|---|
| Yes | | P25325-2 | 3-mercaptopyruvate sulfurtransferase;Sulfurtransferase | MPST | 19,789,000 | 0 | 19,789,000 | 0 | 0 | 0 | 0 | 2 |
| Yes | | A0A3B3ISY1 | Tissue factor pathway inhibitor 2 | TFPI2 | 19,686,000 | 8,176,900 | 0 | 0 | 8,803,000 | 2,706,500 | 0 | 2 |
| Yes | | Q16698-2 | 2,4-dienoyl-CoA reductase, mitochondrial | DECR1 | 19,661,000 | 0 | 19,661,000 | 0 | 0 | 0 | 0 | 2 |
| Yes | | C9JV37 | Prothrombin;Activation peptide fragment 1 | F2 | 19,349,000 | 0 | 0 | 0 | 0 | 19,349,000 | 0 | 1 |
| Yes | | A0A6Q8PFC6 | Glycine amidinotransferase, mitochondrial | GATM | 18,900,000 | 0 | 18,900,000 | 0 | 0 | 0 | 0 | 1 |
| Yes | | A0A075B6H9 | Immunoglobulin lambda variable 4-69 | IGLV4-69 | 18,184,000 | 18,184,000 | 0 | 0 | 0 | 0 | 0 | 1 |
| Yes | | V9GYG0 | ADP/ATP translocase 3;ADP/ATP translocase 3, N-terminally processed | SLC25A4 | 17,956,000 | 0 | 0 | 6,886,600 | 4,931,600 | 0 | 6,137,900 | 1 |
| Yes | | A0A1W2PP35 | Heterogeneous nuclear ribonucleoprotein U | HNRNPU | 17,783,000 | 0 | 0 | 17,783,000 | 0 | 0 | 0 | 3 |
| Yes | | E5RGW4 | Nucleophosmin | NPM1 | 17,763,000 | 0 | 0 | 0 | 0 | 17,763,000 | 0 | 1 |
| Yes | | J3QRN2 | Beta-2-glycoprotein 1 | APOH | 17,755,000 | 0 | 8,891,500 | 0 | 0 | 0 | 8,863,300 | 1 |
| | | E9PRN7 | 3 beta-hydroxysteroid dehydrogenase/Delta 5-->4-isomerase type 2 | HSD3B1 | 17,661,000 | 0 | 0 | 4,061,200 | 6,881,400 | 0 | 6,718,400 | 1 |
| Yes | | P52758 | Ribonuclease UK114 | HRSP12 | 16,603,000 | 0 | 16,603,000 | 0 | 0 | 0 | 0 | 1 |
| Yes | | Q15084-3 | Protein disulfide-isomerase A6 | PDIA6 | 16,371,000 | 0 | 10,683,000 | 0 | 0 | 5,687,900 | 0 | 1 |
| Yes | | Q5TF55 | Delta-1-pyrroline-5-carboxylate dehydrogenase, mitochondrial | ALDH4A1 | 16,343,000 | 0 | 16,343,000 | 0 | 0 | 0 | 0 | 1 |
| Yes | | H0Y4K8 | Fibronectin;Anastellin;Ugl-Y1;Ugl-Y2;Ugl-Y3 | FN1 | 16,129,000 | 2,290,000 | 0 | 7,354,100 | 0 | 0 | 6,485,400 | 1 |
| Yes | | A0A494C1T2 | C-1-tetrahydrofolate synthase, cytoplasmic | MTHFD1 | 15,674,000 | 0 | 15,674,000 | 0 | 0 | 0 | 0 | 3 |
| | | E7EPM6 | Long-chain-fatty-acid--CoA ligase 1 | ACSL1 | 15,503,000 | 0 | 15,503,000 | 0 | 0 | 0 | 0 | 2 |
| Yes | | H0YJF9 | Dihydrolipoyllysine-residue succinyltransferase/2-oxoglutarate dehydrogen | DLST | 14,869,000 | 0 | 0 | 11,035,000 | 0 | 3,833,400 | 0 | 2 |
| Yes | | Q14134-2 | Tripartite motif-containing protein 29 | TRIM29 | 14,797,000 | 0 | 0 | 0 | 0 | 14,797,000 | 0 | 2 |
| Yes | | P18206-2 | Vinculin | VCL | 14,766,000 | 0 | 0 | 0 | 0 | 0 | 14,766,000 | 1 |
| | Yes | D6R9P3 | Heterogeneous nuclear ribonucleoprotein A/B | HNRNPAB | 14,544,000 | 0 | 0 | 14,544,000 | 0 | 0 | 0 | 3 |
| Yes | | P10696 | Alkaline phosphatase, placental-like;Alkaline phosphatase, placental type | ALPPL2 | 14,464,000 | 14,464,000 | 0 | 0 | 0 | 0 | 0 | 1 |
| Yes | | H3BRG4 | Cytochrome b-c1 complex subunit 2, mitochondrial | UQCRC2 | 14,346,000 | 0 | 14,346,000 | 0 | 0 | 0 | 0 | 2 |
| Yes | | C9JRL4 | Malate dehydrogenase;Malate dehydrogenase, cytoplasmic | MDH1 | 14,124,000 | 0 | 14,124,000 | 0 | 0 | 0 | 0 | 1 |
| Yes | | P62258 | 14-3-3 protein epsilon | YWHAE | 14,037,000 | 0 | 5,850,000 | 0 | 0 | 0 | 8,187,500 | 2 |
| Yes | | D6RFG5 | Annexin;Annexin A3 | ANXA3 | 13,723,000 | 0 | 0 | 0 | 0 | 13,723,000 | 0 | 1 |
| Yes | | O60701-3 | UDP-glucose 6-dehydrogenase | UGDH | 13,415,000 | 0 | 13,415,000 | 0 | 0 | 0 | 0 | 2 |
| Yes | | B0YIW2 | Apolipoprotein C-III | APOC3 | 13,244,000 | 0 | 0 | 0 | 0 | 13,244,000 | 0 | 1 |
| Yes | | A0A0G2JQH2 | 40S ribosomal protein S18 | RPS18 | 13,172,000 | 0 | 0 | 0 | 0 | 13,172,000 | 0 | 1 |
| Yes | | P49189 | 4-trimethylaminobutyraldehyde dehydrogenase | ALDH9A1 | 13,161,000 | 0 | 13,161,000 | 0 | 0 | 0 | 0 | 1 |
| Yes | | P11277-3 | Spectrin beta chain, erythrocytic | SPTB | 13,077,000 | 0 | 13,077,000 | 0 | 0 | 0 | 0 | 3 |
| Yes | | Q9HC84 | Mucin-5B | MUC5B | 12,990,000 | 0 | 12,990,000 | 0 | 0 | 0 | 0 | 2 |
| Yes | | P46783 | 40S ribosomal protein S10;Putative 40S ribosomal protein S10-like | RPS10 | 12,790,000 | 0 | 6,330,400 | 0 | 0 | 6,459,400 | 0 | 1 |
| Yes | | P04080 | Cystatin-B | CSTB | 12,717,000 | 7,164,000 | 0 | 5,552,700 | 0 | 0 | 0 | 1 |
| Yes | | F6RFD5 | Destrin | DSTN | 12,619,000 | 0 | 12,619,000 | 0 | 0 | 0 | 0 | 1 |
| Yes | | P25786 | Proteasome subunit alpha type-1;Proteasome subunit alpha type | PSMA1 | 12,242,000 | 0 | 8,949,000 | 3,292,900 | 0 | 0 | 0 | 2 |
| Yes | | P16157-14 | Ankyrin-1 | ANK1 | 12,173,000 | 0 | 12,173,000 | 0 | 0 | 0 | 0 | 2 |
| Yes | | A0A087WT59 | Transthyretin | TTR | 11,910,000 | 0 | 0 | 0 | 0 | 11,910,000 | 0 | 2 |
| Yes | | E9PK47 | Alpha-1,4 glucan phosphorylase;Glycogen phosphorylase, liver form | PYGL | 11,862,000 | 0 | 11,862,000 | 0 | 0 | 0 | 0 | 2 |
| | | P02808 | Statherin | STATH | 11,823,000 | 11,823,000 | 0 | 0 | 0 | 0 | 0 | 1 |
| Yes | | P08246 | Neutrophil elastase | ELANE | 11,630,000 | 0 | 11,630,000 | 0 | 0 | 0 | 0 | 1 |
| | | P52907 | F-actin-capping protein subunit alpha-1 | CAPZA1 | 11,591,000 | 0 | 0 | 0 | 0 | 11,591,000 | 0 | 1 |
| | | C9JA05 | Immunoglobulin J chain | JCHAIN | 11,558,000 | 0 | 0 | 0 | 0 | 11,558,000 | 0 | 1 |
| Yes | | Q9UG54 | Mitogen-activated protein kinase kinase kinase 7 | DKFZp586F0420 | 11,510,000 | 11,510,000 | 0 | 0 | 0 | 0 | 0 | 1 |
| Yes | | P45954-2 | Short/branched chain specific acyl-CoA dehydrogenase, mitochondrial | ACADSB | 11,392,000 | 0 | 11,392,000 | 0 | 0 | 0 | 0 | 1 |
| Yes | | B5MD38 | Trifunctional enzyme subunit beta, mitochondrial;3-ketoacyl-CoA thiolase | HADHB | 11,328,000 | 0 | 11,328,000 | 0 | 0 | 0 | 0 | 1 |
| Yes | | P25815 | Protein S100-P | S100P | 11,215,000 | 0 | 0 | 0 | 0 | 0 | 11,215,000 | 1 |
| Yes | | P02549-2 | Spectrin alpha chain, erythrocytic 1 | SPTA1 | 10,654,000 | 0 | 10,654,000 | 0 | 0 | 0 | 0 | 2 |
| Yes | | H0YAG8 | Alcohol dehydrogenase class-3 | ADH5 | 10,551,000 | 0 | 10,551,000 | 0 | 0 | 0 | 0 | 1 |
| | | A0A087WW87 | Ig kappa chain V-II region FR;Ig kappa chain V-II region Cum | IGKV2-40 | 10,538,000 | 0 | 0 | 0 | 0 | 10,538,000 | 0 | 1 |
| Yes | | B1AKI5 | Muscleblind-like protein 3;Muscleblind-like protein 2 | MBNL3 | 10,394,000 | 0 | 10,394,000 | 0 | 0 | 0 | 0 | 2 |
| Yes | | B1AKQ8 | Guanine nucleotide-binding protein G(I)/G(S)/G(T) subunit beta-1 | GNB1 | 10,354,000 | 0 | 0 | 0 | 0 | 0 | 10,354,000 | 1 |
| Yes | | A0A0J9YXZ5 | Ras GTPase-activating-like protein IQGAP1 | IQGAP1 | 10,328,000 | 0 | 0 | 0 | 0 | 10,328,000 | 0 | 2 |
| Yes | | P13716-2 | Delta-aminolevulinic acid dehydratase | ALAD | 10,259,000 | 0 | 10,259,000 | 0 | 0 | 0 | 0 | 2 |
| Yes | | Q13404-8 | Ubiquitin-conjugating enzyme E2 variant 1 | UBE2V1 | 9,964,900 | 0 | 9,964,900 | 0 | 0 | 0 | 0 | 1 |
| Yes | | M0R0Y6 | Heterogeneous nuclear ribonucleoprotein M | HNRNPM | 9,627,300 | 0 | 0 | 0 | 0 | 9,627,300 | 0 | 1 |
| | | A0A7I2V699 | Nucleolin | NCL | 9,473,600 | 0 | 0 | 0 | 0 | 9,473,600 | 0 | 1 |
| | | P05387 | 60S acidic ribosomal protein P2 | RPLP2 | 9,359,400 | 3,670,500 | 0 | 0 | 0 | 5,688,900 | 0 | 2 |
| Yes | | Q06520 | Bile salt sulfotransferase | SULT2A1 | 9,347,800 | 0 | 9,347,800 | 0 | 0 | 0 | 0 | 1 |
| Yes | | P21399 | Cytoplasmic aconitate hydratase | ACO1 | 9,273,300 | 0 | 9,273,300 | 0 | 0 | 0 | 0 | 1 |
| | | P27824-3 | Calnexin | CANX | 9,232,100 | 0 | 0 | 0 | 0 | 9,232,100 | 0 | 2 |
| Yes | | A0A0C4DGA2 | Enoyl-CoA delta isomerase 2, mitochondrial | ECI2 | 9,217,600 | 0 | 9,217,600 | 0 | 0 | 0 | 0 | 1 |
| | | A0A140TA58 | Keratin-associated protein 9-9 | KRTAP9-9 | 9,046,100 | 0 | 9,046,100 | 0 | 0 | 0 | 0 | 2 |
| Yes | | J3KRG2 | Gasdermin-A | GSDMA | 8,582,300 | 0 | 3,803,000 | 0 | 0 | 0 | 4,779,300 | 2 |
| Yes | | F5GZP6 | Liprin-beta-1 | PPFIBP1 | 8,376,800 | 8,376,800 | 0 | 0 | 0 | 0 | 0 | 1 |
| Yes | | P32926 | Desmoglein-3 | DSG3 | 8,254,400 | 0 | 0 | 0 | 0 | 8,254,400 | 0 | 2 |
| Yes | | A0A0G2JMB2 | Ig alpha-2 chain C region | IGHA2 | 7,976,600 | 0 | 7,976,600 | 0 | 0 | 0 | 0 | 5 |
| Yes | | M0QY85 | Tubulin beta-4A chain;Tubulin beta-4B chain | TUBB4A | 7,933,700 | 0 | 7,933,700 | 0 | 0 | 0 | 0 | 2 |
| Yes | | Q14117 | Dihydropyrimidinase | DPYS | 7,893,100 | 0 | 7,893,100 | 0 | 0 | 0 | 0 | 3 |
| | | E9PE82 | Short-chain specific acyl-CoA dehydrogenase, mitochondrial | ACADS | 7,313,700 | 0 | 7,313,700 | 0 | 0 | 0 | 0 | 1 |
| | | F2Z2W8 | Selenium-binding protein 1 | SELENBP1 | 6,229,500 | 0 | 6,229,500 | 0 | 0 | 0 | 0 | 2 |
| Yes | | Q13011 | Delta(3,5)-Delta(2,4)-dienoyl-CoA isomerase, mitochondrial | ECH1 | 5,760,800 | 0 | 5,760,800 | 0 | 0 | 0 | 0 | 1 |
| | | Q9UBG3 | Cornulin | CRNN | 5,651,400 | 0 | 0 | 5,651,400 | 0 | 0 | 0 | 1 |
| | | Q08257-2 | Quinone oxidoreductase | CRYZ | 5,461,300 | 0 | 5,461,300 | 0 | 0 | 0 | 0 | 1 |
| Yes | | P28838-2 | Cytosol aminopeptidase | LAP3 | 5,135,200 | 0 | 5,135,200 | 0 | 0 | 0 | 0 | 1 |
| Yes | | A8MUD9 | 60S ribosomal protein L7 | RPL7 | 4,984,000 | 4,984,000 | 0 | 0 | 0 | 0 | 0 | 1 |
| | | E9PEB5 | Far upstream element-binding protein 1 | FUBP1 | 4,190,600 | 0 | 0 | 4,190,600 | 0 | 0 | 0 | 2 |
| Yes | | P09651 | Heterogeneous nuclear ribonucleoprotein A1 | HNRNPA1 | 3,752,800 | 3,752,800 | 0 | 0 | 0 | 0 | 0 | 2 |
| | | P22314-2 | Ubiquitin-like modifier-activating enzyme 1 | UBA1 | 3,748,100 | 0 | 3,748,100 | 0 | 0 | 0 | 0 | 1 |
| Yes | | Q14032 | Bile acid-CoA:amino acid N-acyltransferase | BAAT | 3,451,500 | 0 | 3,451,500 | 0 | 0 | 0 | 0 | 1 |
| Yes | | G3V5K1 | Protein transport protein Sec23A | SEC23A | 2,912,600 | 0 | 0 | 0 | 0 | 2,912,600 | 0 | 1 |
| | | H7C4C8 | T-complex protein 1 subunit theta | CCT8 | 2,848,600 | 0 | 2,848,600 | 0 | 0 | 0 | 0 | 1 |
| | | E9PBS1 | Multifunctional protein ADE2;Phosphoribosylaminoimidazole carboxylase | PAICS | 2,615,600 | 0 | 2,615,600 | 0 | 0 | 0 | 0 | 1 |

**Supplemental Table 3**
**Proteins identified in PICALM immunoprecipitations.**
Placental extracts U1, R4, Q1, Q4, O1, and N3 were immunoprecipitated in one experiment.

| Majority protein ID | Protein | Gene | Total Intensity | Intensity PICALM U1 | Intensity PICALM R4 | Intensity PICALM Q1 | Intensity PICALM Q4 | Intensity PICALM O1 | Intensity PICALM N3 | Peptides |
|---|---|---|---|---|---|---|---|---|---|---|
| P01857 | Ig gamma-1 chain C region | IGHG1 | 48,398,000,000 | 197,890,000 | 13,091,000,000 | 789,180,000 | 14,626,000,000 | 12,977,000,000 | 6,715,700,000 | 23 |
| Q13492 | Phosphatidylinositol-binding clathrin assembly prote | PICALM | 18,858,000,000 | 901,110,000 | 5,271,000,000 | 657,650,000 | 3,743,800,000 | 4,066,800,000 | 4,217,400,000 | 35 |
| P50995-2 | Annexin A11 | ANXA11 | 15,851,000,000 | 139,870,000 | 4,529,400,000 | 433,220,000 | 3,795,300,000 | 3,270,300,000 | 3,682,800,000 | 37 |
| P20073-2 | Annexin A7 | ANXA7 | 9,130,600,000 | 666,020,000 | 2,765,200,000 | 238,940,000 | 1,535,100,000 | 2,049,900,000 | 1,875,500,000 | 32 |
| A0A286YES1 | Ig gamma-3 chain C region | IGHG3 | 4,336,300,000 | 33,112,000 | 1,106,600,000 | 53,173,000 | 927,810,000 | 812,970,000 | 1,402,600,000 | 20 |
| A0A7I2V378 | Laminin subunit beta-1 | LAMB1 | 3,056,100,000 | 0 | 3,056,100,000 | 0 | 0 | 0 | 0 | 1 |
| E7EVS6 | Actin, cytoplasmic 1;Actin, cytoplasmic 1, N-termina | ACTB | 2,600,000,000 | 74,816,000 | 1,747,800,000 | 140,870,000 | 230,680,000 | 217,870,000 | 187,980,000 | 19 |
| P01834 | Ig kappa chain C region | IGKC | 2,165,500,000 | 19,359,000 | 221,050,000 | 1,391,900,000 | 195,150,000 | 232,100,000 | 105,920,000 | 9 |
| A0A286YFJ8 | Ig gamma-4 chain C region | IGHG4 | 1,609,600,000 | 0 | 218,830,000 | 22,468,000 | 138,990,000 | 428,050,000 | 801,270,000 | 17 |
| P0DOY3 | Immunoglobulin lambda constant 3 | IGLC3 | 1,591,000,000 | 2,681,900 | 60,706,000 | 741,050,000 | 302,850,000 | 240,510,000 | 243,250,000 | 9 |
| A0A286YEY1 | Ig alpha-1 chain C region | IGHA1 | 1,335,500,000 | 23,359,000 | 584,930,000 | 130,660,000 | 252,550,000 | 139,020,000 | 204,990,000 | 14 |
| Q9Y6R7 | IgGFc-binding protein | FCGBP | 1,098,700,000 | 9,162,000 | 128,730,000 | 9,654,400 | 38,684,000 | 824,150,000 | 88,293,000 | 30 |
| P01624 | Ig kappa chain V-III region POM | IGKV3D-15 | 1,060,200,000 | 0 | 124,770,000 | 613,850,000 | 116,590,000 | 205,020,000 | 0 | 2 |
| P31943 | Heterogeneous nuclear ribonucleoprotein H;Heterog | HNRNPH1 | 1,047,700,000 | 10,293,000 | 217,040,000 | 24,254,000 | 289,540,000 | 295,340,000 | 211,190,000 | 11 |
| P08670 | Vimentin | VIM | 951,490,000 | 6,378,400 | 298,510,000 | 5,089,400 | 72,211,000 | 358,520,000 | 210,780,000 | 26 |
| H0YEF7 | Phosphatidylinositol-binding clathrin assembly prote | PICALM | 874,480,000 | 16,602,000 | 212,520,000 | 57,324,000 | 467,540,000 | 68,277,000 | 52,211,000 | 14 |
| E9PQV5 | Coiled-coil domain-containing protein 37 | CCDC37 | 870,490,000 | 0 | 0 | 870,490,000 | 0 | 0 | 0 | 1 |
| A0A4W8ZXM2 | Immunoglobulin heavy variable 3-72 | IGHV3-72 | 808,240,000 | 44,918,000 | 282,930,000 | 12,296,000 | 137,660,000 | 204,230,000 | 126,190,000 | 3 |
| P05783 | Keratin, type I cytoskeletal 18 | KRT18 | 760,020,000 | 0 | 231,740,000 | 8,635,500 | 185,390,000 | 187,570,000 | 146,680,000 | 17 |
| P07355 | Annexin A2;Annexin;Putative annexin A2-like protei | ANXA2 | 742,370,000 | 6,496,700 | 256,760,000 | 55,166,000 | 54,726,000 | 196,500,000 | 172,720,000 | 15 |
| P01871 | Ig mu chain C region | IGHM | 722,270,000 | 105,950,000 | 12,770,000 | 0 | 95,422,000 | 155,950,000 | 352,180,000 | 18 |
| P31942-2 | Heterogeneous nuclear ribonucleoprotein H3 | HNRNPH3 | 681,640,000 | 0 | 133,900,000 | 68,508,000 | 133,680,000 | 231,950,000 | 113,610,000 | 9 |
| Q8NDC0 | MAPK-interacting and spindle-stabilizing protein-like | MAPK1IP1L | 497,400,000 | 15,599,000 | 39,220,000 | 243,740,000 | 59,063,000 | 32,791,000 | 106,990,000 | 1 |
| P51512-2 | Matrix metalloproteinase-16 | MMP16 | 429,540,000 | 0 | 178,440,000 | 27,390,000 | 0 | 223,720,000 | 0 | 1 |
| H0Y8G5 | Heterogeneous nuclear ribonucleoprotein D0 | HNRNPD | 409,120,000 | 0 | 31,290,000 | 27,715,000 | 95,052,000 | 151,740,000 | 103,320,000 | 5 |
| E7ETU5 | RNA-binding motif, single-stranded-interacting prote | RBMS1 | 391,920,000 | 5,970,500 | 68,667,000 | 17,784,000 | 143,760,000 | 44,979,000 | 110,760,000 | 7 |
| Q8WZ42 | Titin | TTN | 387,820,000 | 0 | 0 | 0 | 75,928,000 | 210,050,000 | 101,840,000 | 1 |
| P02675 | Fibrinogen beta chain;Fibrinopeptide B;Fibrinogen b | FGB | 348,580,000 | 0 | 343,520,000 | 2,249,600 | 0 | 0 | 2,806,500 | 16 |
| A0A0D9SF16 | Methyl-CpG-binding domain protein 5 | MBD5 | 346,080,000 | 0 | 0 | 0 | 346,080,000 | 0 | 0 | 1 |
| O14979-3 | Heterogeneous nuclear ribonucleoprotein D-like | HNRNPDL | 334,140,000 | 4,097,000 | 84,425,000 | 6,682,100 | 92,722,000 | 38,095,000 | 108,120,000 | 6 |
| P98082 | Disabled homolog 2 | DAB2 | 308,620,000 | 30,904,000 | 53,571,000 | 22,044,000 | 29,573,000 | 79,395,000 | 93,129,000 | 8 |
| A0A286YEY4 | Ig gamma-2 chain C region | IGHG2 | 295,690,000 | 3,630,700 | 140,820,000 | 13,340,000 | 30,486,000 | 32,731,000 | 74,683,000 | 15 |
| P68871 | Hemoglobin subunit beta;LVV-hemorphin-7;Spinorp | HBB | 289,630,000 | 17,601,000 | 25,216,000 | 58,392,000 | 73,808,000 | 70,816,000 | 43,798,000 | 4 |
| P11021 | 78 kDa glucose-regulated protein | HSPA5 | 278,720,000 | 66,634,000 | 8,224,700 | 0 | 44,577,000 | 12,509,000 | 146,770,000 | 14 |
| P04083 | Annexin A1;Annexin | ANXA1 | 264,550,000 | 7,343,100 | 128,430,000 | 38,085,000 | 12,426,000 | 24,473,000 | 53,792,000 | 13 |
| P01594 | Ig kappa variable 1-33 | IGKV1-33 | 258,450,000 | 2,181,900 | 6,309,800 | 167,650,000 | 34,900,000 | 29,177,000 | 18,233,000 | 2 |
| O43866 | CD5 antigen-like | CD5L | 258,210,000 | 0 | 64,662,000 | 0 | 27,301,000 | 135,430,000 | 30,812,000 | 5 |
| F8VV32 | Lysozyme;Lysozyme C | LYZ | 201,490,000 | 13,865,000 | 0 | 33,772,000 | 58,974,000 | 48,689,000 | 46,193,000 | 3 |
| P21980-2 | Protein-glutamine gamma-glutamyltransferase 2 | TGM2 | 182,780,000 | 21,051,000 | 6,206,000 | 155,520,000 | 0 | 0 | 0 | 4 |
| P01615 | Immunoglobulin kappa varibable 2D-28 | IGKV2D-28 | 157,840,000 | 0 | 0 | 56,727,000 | 2,124,500 | 1,244,100 | 97,746,000 | 1 |
| Q5T749 | Keratinocyte proline-rich protein | KPRP | 156,670,000 | 0 | 14,536,000 | 42,583,000 | 12,523,000 | 41,980,000 | 45,045,000 | 8 |
| Q5T7N2 | LINE-1 type transposase domain-containing protein | L1TD1 | 147,570,000 | 0 | 33,515,000 | 51,668,000 | 0 | 0 | 62,386,000 | 2 |
| A0A6Q8PGK1 | Heat shock protein beta-1 | HSPB1 | 140,170,000 | 5,102,400 | 5,175,100 | 116,740,000 | 0 | 9,140,400 | 4,009,100 | 4 |
| Q02413 | Desmoglein-1 | DSG1 | 139,520,000 | 4,467,600 | 13,159,000 | 21,449,000 | 21,775,000 | 52,035,000 | 26,635,000 | 9 |
| F8VVB9 | Tubulin alpha-1B chain;Tubulin alpha-1A chain;Tub | TUBA1B | 130,470,000 | 4,960,800 | 53,076,000 | 11,800,000 | 10,998,000 | 17,150,000 | 32,482,000 | 4 |
| A0A6Q8PFJ0 | Prelamin-A/C;Lamin-A/C | LMNA | 126,810,000 | 51,770,000 | 36,048,000 | 0 | 9,173,200 | 5,398,800 | 24,420,000 | 7 |
| P69905 | Hemoglobin subunit alpha | HBA1 | 120,580,000 | 8,819,100 | 32,928,000 | 0 | 19,697,000 | 31,037,000 | 28,103,000 | 3 |
| P02671 | Fibrinogen alpha chain;Fibrinopeptide A;Fibrinogen | FGA | 119,010,000 | 5,765,800 | 89,186,000 | 13,712,000 | 0 | 1,589,500 | 8,753,800 | 6 |
| A0A0B4J231 | Immunoglobulin lambda-like polypeptide 5;Ig lambd | IGLL5 | 113,780,000 | 0 | 823,960 | 0 | 51,884,000 | 45,566,000 | 15,503,000 | 7 |
| Q9HAU0-6 | Pleckstrin homology domain-6 | PLEKHA6 | 100,890,000 | 0 | 9,826,200 | 91,063,000 | 0 | 0 | 0 | 1 |
| Q92945 | Far upstream element-binding protein 2 | KHSRP | 96,813,000 | 25,823,000 | 0 | 0 | 5,257,900 | 16,437,000 | 49,295,000 | 8 |
| R4GNB1 | Acyl-CoA synthetase family member 4 | AASDH | 94,959,000 | 0 | 6,802,400 | 6,567,300 | 14,507,000 | 47,725,000 | 19,358,000 | 1 |
| C9JEU5 | Fibrinogen gamma chain | FGG | 90,470,000 | 63,046,000 | 15,905,000 | 0 | 10,380,000 | 0 | 1,139,700 | 7 |
| P04406 | Glyceraldehyde-3-phosphate dehydrogenase | GAPDH | 89,398,000 | 4,504,400 | 74,117,000 | 3,593,000 | 1,898,700 | 5,284,800 | 0 | 6 |
| K7ERX7 | ATP synthase subunit alpha, mitochondrial | ATP5A1 | 87,648,000 | 0 | 49,222,000 | 0 | 0 | 20,992,000 | 17,434,000 | 5 |
| E9PPG9 | mRNA export factor | RAE1 | 87,447,000 | 0 | 0 | 0 | 58,237,000 | 2,416,200 | 26,794,000 | 4 |
| P30101 | Protein disulfide-isomerase A3 | PDIA3 | 86,682,000 | 0 | 34,607,000 | 0 | 0 | 17,446,000 | 34,628,000 | 8 |
| K7EQ02 | DAZ-associated protein 1 | DAZAP1 | 83,974,000 | 0 | 11,234,000 | 0 | 42,826,000 | 23,140,000 | 6,774,000 | 2 |
| Q01469 | Fatty acid-binding protein, epidermal | FABP5 | 79,112,000 | 0 | 8,010,100 | 12,771,000 | 28,053,000 | 0 | 30,278,000 | 1 |
| Q01085 | Nucleolysin TIAR;Nucleolysin TIA-1 isoform p40 | TIAL1 | 77,006,000 | 0 | 15,592,000 | 0 | 14,334,000 | 18,441,000 | 28,639,000 | 4 |
| P21397-2 | Amine oxidase [flavin-containing] A;Amine oxidase | MAOA | 76,723,000 | 0 | 33,332,000 | 0 | 16,437,000 | 0 | 26,954,000 | 4 |
| A6NHN2 | Roquin-2 | RC3H2 | 74,367,000 | 0 | 0 | 50,159,000 | 0 | 8,880,000 | 15,328,000 | 1 |
| P14866 | Heterogeneous nuclear ribonucleoprotein L | HNRNPL | 68,836,000 | 0 | 0 | 0 | 0 | 0 | 68,836,000 | 7 |
| Q5JPU3 | Pyruvate dehydrogenase E1 component subunit alp | PDHA1 | 66,908,000 | 0 | 12,367,000 | 0 | 31,262,000 | 13,254,000 | 10,024,000 | 3 |
| Q8IUC1 | Keratin-associated protein 11-1 | KRTAP11-1 | 65,493,000 | 0 | 18,492,000 | 28,950,000 | 0 | 18,051,000 | 0 | 3 |
| Q5JP53 | Tubulin beta chain | TUBB | 64,164,000 | 3,856,400 | 37,532,000 | 2,310,200 | 7,223,200 | 4,449,300 | 8,792,800 | 3 |
| C9J2I0 | Arf-GAP domain and FG repeat-containing protein 1 | AGFG1 | 59,976,000 | 4,759,100 | 12,457,000 | 0 | 0 | 18,719,000 | 24,041,000 | 1 |
| P19474 | E3 ubiquitin-protein ligase TRIM21 | TRIM21 | 58,811,000 | 0 | 6,681,800 | 0 | 0 | 29,267,000 | 22,862,000 | 5 |
| D6R9P3 | Heterogeneous nuclear ribonucleoprotein A/B | HNRNPAB | 58,548,000 | 0 | 7,571,700 | 2,186,500 | 9,640,700 | 28,004,000 | 11,145,000 | 4 |
| Q5T1M5 | FK506-binding protein 15 | FKBP15 | 58,396,000 | 11,484,000 | 0 | 0 | 14,598,000 | 0 | 32,314,000 | 4 |
| E9PGY2 | Dynein assembly factor 5, axonemal | DNAAF5 | 56,016,000 | 0 | 0 | 0 | 20,626,000 | 25,326,000 | 10,064,000 | 1 |
| P63261 | Actin, cytoplasmic 2;Actin, cytoplasmic 2, N-termina | ACTG1 | 55,103,000 | 0 | 29,965,000 | 6,910,800 | 6,077,800 | 5,127,100 | 7,022,400 | 19 |
| Q9NZT1 | Calmodulin-like protein 5 | CALML5 | 54,136,000 | 0 | 7,928,200 | 10,694,000 | 0 | 27,905,000 | 7,608,500 | 1 |
| C9JZN1 | Guanine nucleotide-binding protein G(I)/G(S)/G(T) s | GNB2 | 53,184,000 | 0 | 0 | 45,048,000 | 8,135,900 | 0 | 0 | 4 |
| A0A7I2YQK6 | 60 kDa heat shock protein, mitochondrial | HSPD1 | 50,218,000 | 1,514,400 | 15,195,000 | 0 | 15,571,000 | 6,895,200 | 11,042,000 | 3 |
| P31040-2 | Succinate dehydrogenase [ubiquinone] flavoprotein | SDHA | 49,094,000 | 0 | 23,561,000 | 0 | 3,205,300 | 15,958,000 | 6,369,600 | 5 |
| A0A7I2V2R3 | Heterogeneous nuclear ribonucleoprotein A3 | HNRNPA3 | 48,903,000 | 0 | 15,655,000 | 0 | 7,296,200 | 11,237,000 | 14,714,000 | 2 |
| Q8IWB6-3 | Inactive serine/threonine-protein kinase TEX14 | TEX14 | 48,249,000 | 0 | 0 | 0 | 0 | 21,743,000 | 26,507,000 | 1 |
| P68371 | Tubulin beta-4B chain;Tubulin beta-4A chain | TUBB4B | 48,032,000 | 0 | 39,150,000 | 0 | 4,137,600 | 0 | 4,744,800 | 3 |
| H0YFX9 | Histone H2A;Histone H2A type 1-J;Histone H2A typ | H2AFJ | 45,901,000 | 12,453,000 | 7,861,100 | 20,288,000 | 0 | 0 | 5,298,900 | 1 |
| Q8IUC0 | Keratin-associated protein 13-1;Keratin-associated p | KRTAP13-1 | 44,512,000 | 0 | 7,315,500 | 7,365,500 | 0 | 29,831,000 | 0 | 1 |
| A0A087WVQ6 | Clathrin heavy chain;Clathrin heavy chain 1 | CLTC | 44,504,000 | 21,103,000 | 0 | 0 | 0 | 0 | 23,400,000 | 3 |
| P63267 | Actin, gamma-enteric smooth muscle;Actin, alpha s | ACTG2 | 44,025,000 | 0 | 23,112,000 | 0 | 0 | 8,310,000 | 12,603,000 | 12 |
| H7BZJ3 | Protein disulfide-isomerase A3 | PDIA3 | 43,257,000 | 4,680,500 | 27,448,000 | 0 | 0 | 11,128,000 | 0 | 3 |
| A0A0B4J1V1 | Ig heavy chain V-III region JON;Ig heavy chain V-III | IGHV3-21 | 43,147,000 | 0 | 9,064,600 | 2,411,600 | 0 | 22,858,000 | 8,812,900 | 2 |
| H7C582 | Integrator complex subunit 1 | INTS1 | 42,661,000 | 0 | 0 | 42,661,000 | 0 | 0 | 0 | 1 |
| Q9BYR6 | Keratin-associated protein 3-3 | KRTAP3-3 | 40,458,000 | 0 | 40,458,000 | 0 | 0 | 0 | 0 | 2 |
| Q13151 | Heterogeneous nuclear ribonucleoprotein A0 | HNRNPA0 | 39,870,000 | 0 | 0 | 5,577,800 | 21,067,000 | 9,442,300 | 3,783,300 | 1 |
| H0Y6B2 | Sushi domain-containing protein 1 | SUSD1 | 38,095,000 | 0 | 0 | 22,678,000 | 8,950,800 | 6,466,400 | 0 | 1 |
| A0A1B0GVP4 | Ligand-dependent nuclear receptor corepressor | LCORL | 36,335,000 | 0 | 16,645,000 | 0 | 9,411,000 | 0 | 10,278,000 | 1 |
| P14923 | Junction plakoglobin | JUP | 35,503,000 | 0 | 0 | 4,915,000 | 0 | 21,240,000 | 9,347,600 | 6 |
| P01023 | Alpha-2-macroglobulin | A2M | 35,281,000 | 0 | 0 | 0 | 18,291,000 | 0 | 16,990,000 | 2 |
| A0A7I2V3H3 | Putative elongation factor 1-alpha-like 3;Elongation | EEF1A1 | 34,691,000 | 0 | 20,615,000 | 0 | 0 | 6,899,500 | 7,176,500 | 3 |
| I3L0W5 | 14-3-3 protein epsilon | YWHAE | 34,151,000 | 0 | 4,124,200 | 16,649,000 | 3,202,500 | 4,882,900 | 5,292,400 | 1 |
| P02787 | Serotransferrin | TF | 33,379,000 | 5,509,000 | 0 | 4,698,000 | 8,538,800 | 1,682,500 | 12,951,000 | 3 |
| Q08554-2 | Desmocollin-1 | DSC1 | 31,458,000 | 0 | 0 | 2,709,100 | 0 | 22,304,000 | 6,444,900 | 2 |
| E5RGE1 | 14-3-3 protein zeta/delta | YWHAZ | 31,118,000 | 0 | 0 | 31,118,000 | 0 | 0 | 0 | 2 |
| P06576 | ATP synthase subunit beta, mitochondrial;ATP synt | ATP5B | 31,113,000 | 0 | 31,113,000 | 0 | 0 | 0 | 0 | 3 |
| P17931 | Galectin-3;Galectin | LGALS3 | 30,883,000 | 0 | 0 | 30,883,000 | 0 | 0 | 0 | 3 |
| C9JA05 | Immunoglobulin J chain | JCHAIN | 29,005,000 | 6,597,900 | 8,495,000 | 8,405,600 | 0 | 0 | 5,506,600 | 1 |

| | | | | | | | | | | |
|---|---|---|---|---|---|---|---|---|---|---|
| A0A0G2JRN3 | Alpha-1-antitrypsin;Short peptide from AAT | SERPINA1 | 28,649,000 | 1,644,400 | 2,510,900 | 10,667,000 | 8,084,300 | 1,831,300 | 3,911,300 | 2 |
| P31025 | Lipocalin-1;Putative lipocalin 1-like protein 1 | LCN1 | 28,552,000 | 0 | 0 | 2,845,200 | 0 | 6,027,100 | 19,679,000 | 2 |
| P07237 | Protein disulfide-isomerase | P4HB | 28,383,000 | 0 | 18,574,000 | 0 | 0 | 4,970,100 | 4,837,900 | 3 |
| H0Y5H6 | Ubiquitin-associated protein 2-like | UBAP2L | 25,709,000 | 0 | 0 | 0 | 0 | 8,179,400 | 17,530,000 | 1 |
| Q96CBB | Integrator complex subunit 12 | INTS12 | 25,627,000 | 0 | 0 | 0 | 0 | 25,627,000 | 0 | 1 |
| S4R460 | Ig heavy chain V-III region BRO region DOB | IGHV3OR16-9 | 24,932,000 | 0 | 13,396,000 | 0 | 0 | 6,415,600 | 5,120,200 | 3 |
| Q5JTQ6 | Alpha-catulin | CTNNAL1 | 23,896,000 | 0 | 0 | 17,301,000 | 0 | 6,594,900 | 0 | 1 |
| P07910 | Heterogeneous nuclear ribonucleoproteins C1/C2 | HNRNPC | 23,699,000 | 0 | 15,502,000 | 0 | 8,197,000 | 0 | 0 | 2 |
| E9PEB5 | Far upstream element-binding protein 1 | FUBP1 | 23,687,000 | 0 | 0 | 0 | 0 | 20,589,000 | 3,098,200 | 3 |
| Q07065 | Cytoskeleton-associated protein 4 | CKAP4 | 23,211,000 | 0 | 10,484,000 | 0 | 0 | 7,232,200 | 5,495,600 | 5 |
| Q9UBG3 | Cornulin | CRNN | 23,063,000 | 0 | 0 | 23,063,000 | 0 | 0 | 0 | 3 |
| A0A0C4DH31 | Ig heavy chain V-I region V35 | IGHV1-18 | 22,114,000 | 0 | 7,494,400 | 0 | 0 | 8,305,200 | 6,314,800 | 2 |
| P04843 | Dolichyl-diphosphooligosaccharide--protein glycosyl | RPN1 | 21,647,000 | 0 | 6,872,300 | 0 | 2,481,300 | 3,898,300 | 8,395,600 | 3 |
| A0A0G2JIW1 | Heat shock 70 kDa protein 1A;Heat shock 70 kDa pr | HSPA1A | 21,113,000 | 1,946,300 | 16,283,000 | 0 | 0 | 2,883,300 | 0 | 4 |
| P11142-2 | Heat shock cognate 71 kDa protein | HSPA8 | 20,977,000 | 14,054,000 | 0 | 1,367,200 | 1,373,400 | 4,182,300 | 0 | 6 |
| Q15365 | Poly(rC)-binding protein 1;Poly(rC)-binding protein 3 | PCBP1 | 19,851,000 | 0 | 8,243,700 | 0 | 0 | 7,620,100 | 3,987,300 | 4 |
| P29401 | Transketolase | TKT | 19,791,000 | 2,617,800 | 17,173,000 | 0 | 0 | 0 | 0 | 3 |
| P11166 | Solute carrier family 2, facilitated glucose transporte | SLC2A1 | 19,512,000 | 0 | 19,512,000 | 0 | 0 | 0 | 0 | 1 |
| M0R3F1 | Heterogeneous nuclear ribonucleoprotein U-like pro | HNRNPUL1 | 19,055,000 | 0 | 0 | 0 | 0 | 0 | 19,055,000 | 4 |
| P69892 | Hemoglobin subunit gamma-2;Hemoglobin subunit | HBG2 | 18,869,000 | 8,500,000 | 10,370,000 | 0 | 0 | 0 | 0 | 4 |
| A0A7I2V4I6 | Heterogeneous nuclear ribonucleoproteins A2/B1 | HNRNPA2B1 | 18,704,000 | 0 | 0 | 0 | 0 | 12,162,000 | 6,541,300 | 4 |
| A0A5F9ZH78 | Arginase-1 | ARG1 | 17,366,000 | 0 | 4,850,200 | 0 | 0 | 5,206,700 | 7,309,200 | 2 |
| J3KPS3 | Fructose-bisphosphate aldolase;Fructose-bisphosph | ALDOA | 16,868,000 | 0 | 16,868,000 | 0 | 0 | 0 | 0 | 3 |
| A0A7I2YQY2 | Heterogeneous nuclear ribonucleoprotein A1;Heterc | HNRNPA1 | 16,360,000 | 2,149,600 | 2,573,000 | 0 | 7,993,500 | 3,644,300 | 0 | 1 |
| Q9BYQ3 | Keratin-associated protein 9-3 | KRTAP9-3 | 16,191,000 | 0 | 16,191,000 | 0 | 0 | 0 | 0 | 2 |
| Q9BS26 | Endoplasmic reticulum resident protein 44 | ERP44 | 16,007,000 | 0 | 7,136,300 | 0 | 0 | 7,435,300 | 1,435,800 | 3 |
| P15924-2 | Desmoplakin | DSP | 15,924,000 | 0 | 0 | 6,126,800 | 0 | 9,797,400 | 0 | 5 |
| P05109 | Protein S100-A8;Protein S100-A8, N-terminally proc | S100A8 | 15,760,000 | 828,670 | 4,227,300 | 10,704,000 | 0 | 0 | 0 | 1 |
| E9PMZ8 | T-lymphoma invasion and metastasis-inducing prote | TIAM2 | 15,463,000 | 0 | 0 | 15,463,000 | 0 | 0 | 0 | 1 |
| P01833 | Polymeric immunoglobulin receptor;Secretory comp | PIGR | 15,458,000 | 0 | 11,036,000 | 0 | 0 | 3,208,000 | 1,214,700 | 3 |
| H3BQZ7 | Heterogeneous nuclear ribonucleoprotein U-like pro | HNRNPUL2-BSCL2 | 15,173,000 | 1,669,300 | 0 | 0 | 0 | 0 | 13,503,000 | 1 |
| P55795 | Heterogeneous nuclear ribonucleoprotein H2 | HNRNPH2 | 14,987,000 | 0 | 4,489,200 | 0 | 1,825,300 | 5,079,900 | 3,592,900 | 5 |
| A0A0G2JPP1 | Keratin-associated protein 4-8 | KRTAP4-8 | 14,521,000 | 0 | 14,521,000 | 0 | 0 | 0 | 0 | 1 |
| E9PHT9 | Annexin;Annexin A5 | ANXA5 | 12,946,000 | 0 | 0 | 12,946,000 | 0 | 0 | 0 | 2 |
| A0A6Q8PF87 | Apoptosis-inducing factor 1, mitochondrial | AIFM1 | 12,539,000 | 0 | 12,539,000 | 0 | 0 | 0 | 0 | 1 |
| M0R1R1 | Serine/threonine-protein kinase PAK 4 | PAK4 | 12,509,000 | 0 | 0 | 0 | 0 | 0 | 12,509,000 | 1 |
| P04040 | Catalase | CAT | 12,392,000 | 0 | 3,717,800 | 1,498,500 | 0 | 3,932,900 | 3,243,300 | 1 |
| A0A140TA58 | Keratin-associated protein 9-9;Keratin-associated pr | KRTAP9-9 | 12,125,000 | 0 | 12,125,000 | 0 | 0 | 0 | 0 | 2 |
| A0A0A0MS98 | Band 3 anion transport protein | SLC4A1 | 12,063,000 | 2,108,100 | 0 | 0 | 3,363,800 | 0 | 6,591,400 | 1 |
| P0DP03 | Ig heavy chain V-III region CAM;Ig heavy chain V-II | IGHV3-23 | 11,962,000 | 0 | 0 | 0 | 0 | 7,237,200 | 4,724,800 | 1 |
| P81605 | Dermcidin;Survival-promoting peptide;DCD-1 | DCD | 11,727,000 | 0 | 0 | 7,387,600 | 0 | 4,338,900 | 0 | 2 |
| H0YFA4 | Cysteine-rich protein 2 | CRIP2 | 11,648,000 | 0 | 0 | 7,587,800 | 4,060,200 | 0 | 0 | 2 |
| A0A3B3ITD8 | Nuclear pore complex protein Nup98-Nup96;Nuclea | NUP98 | 11,301,000 | 0 | 0 | 0 | 11,301,000 | 0 | 0 | 2 |
| P14625 | Endoplasmin | HSP90B1 | 11,207,000 | 11,207,000 | 0 | 0 | 0 | 0 | 0 | 4 |
| Q96PK6 | RNA-binding protein 14 | RBM14 | 11,150,000 | 0 | 0 | 0 | 7,074,900 | 0 | 4,075,400 | 1 |
| E7ENL6 | Collagen alpha-3(VI) chain | COL6A3 | 10,671,000 | 10,671,000 | 0 | 0 | 0 | 0 | 0 | 3 |
| F8WBE5 | Transferrin receptor protein 1;Transferrin receptor p | TFRC | 10,642,000 | 3,853,100 | 0 | 0 | 0 | 0 | 6,788,600 | 1 |
| A0A0G2JMB2 | Ig alpha-2 chain C region | IGHA2 | 10,360,000 | 0 | 5,177,500 | 0 | 0 | 2,870,900 | 2,311,700 | 9 |
| A0A7I2YQJ0 | Transitional endoplasmic reticulum ATPase | VCP | 10,078,000 | 10,078,000 | 0 | 0 | 0 | 0 | 0 | 2 |
| B3KVK2 | Guanine nucleotide-binding protein G(I)/G(S)/G(T) s | GNB1 | 9,941,900 | 0 | 0 | 6,870,800 | 0 | 3,071,100 | 0 | 1 |
| Q9BYR3 | Keratin-associated protein 4-4 | KRTAP4-4 | 9,925,900 | 0 | 9,925,900 | 0 | 0 | 0 | 0 | 1 |
| P52597 | Heterogeneous nuclear ribonucleoprotein F;Heterog | HNRNPF | 9,850,000 | 0 | 3,857,200 | 0 | 0 | 0 | 5,992,800 | 3 |
| P01024 | Complement C3;Complement C3 beta chain;C3-bet | C3 | 9,848,400 | 0 | 9,848,400 | 0 | 0 | 0 | 0 | 2 |
| P80723-2 | Brain acid soluble protein 1 | BASP1 | 9,527,100 | 0 | 0 | 0 | 0 | 0 | 9,527,100 | 2 |
| F8W1T6 | RNA-binding motif, single-stranded-interacting prote | RBMS2 | 9,438,700 | 0 | 0 | 0 | 0 | 0 | 9,438,700 | 4 |
| P31944 | Caspase-14;Caspase-14 subunit p17, mature form;( | CASP14 | 9,239,700 | 0 | 0 | 5,161,200 | 0 | 0 | 4,078,600 | 1 |
| P0DP08 | Ig heavy chain V-II region NEWM;Ig heavy chain V- | IGHV4-4 | 9,180,400 | 0 | 0 | 0 | 0 | 5,589,100 | 3,591,300 | 1 |
| H3BU13 | Pyruvate kinase;Pyruvate kinase PKM | PKM | 9,106,300 | 0 | 9,106,300 | 0 | 0 | 0 | 0 | 1 |
| F8WEU2 | ATP-dependent 6-phosphofructokinase, liver type | PFKL | 9,083,300 | 9,083,300 | 0 | 0 | 0 | 0 | 0 | 1 |
| A0A0C4DH25 | Immunoglobulin kappa variable 3D-20 | IGKV3D-20 | 8,934,800 | 0 | 0 | 0 | 0 | 0 | 8,934,800 | 1 |
| P52907 | F-actin-capping protein subunit alpha-1 | CAPZA1 | 8,912,600 | 0 | 8,912,600 | 0 | 0 | 0 | 0 | 1 |
| I3L1P8 | Mitochondrial 2-oxoglutarate/malate carrier protein | SLC25A11 | 8,767,800 | 0 | 0 | 8,767,800 | 0 | 0 | 0 | 1 |
| B4E3S0 | Coronin;Coronin-1C | CORO1C | 8,702,200 | 0 | 4,567,500 | 0 | 0 | 4,134,700 | 0 | 1 |
| F8W6D9 | Sentrin-specific protease 6 | SENP6 | 8,679,600 | 0 | 0 | 0 | 0 | 0 | 8,679,600 | 2 |
| P36957-2 | Dihydrolipoyllysine-residue succinyltransferase com | DLST | 8,276,700 | 0 | 5,934,900 | 0 | 0 | 0 | 2,341,800 | 1 |
| P31930 | Cytochrome b-c1 complex subunit 1, mitochondrial | UQCRC1 | 8,234,200 | 0 | 3,957,100 | 0 | 0 | 0 | 4,277,000 | 2 |
| P02808 | Statherin | STATH | 8,064,100 | 0 | 0 | 8,064,100 | 0 | 0 | 0 | 1 |
| P16403 | Histone H1.2;Histone H1.3 | HIST1H1C | 7,889,100 | 0 | 2,483,600 | 5,405,500 | 0 | 0 | 0 | 3 |
| K7EMF8 | Very long-chain specific acyl-CoA dehydrogenase, r | ACADVL | 7,844,000 | 0 | 7,844,000 | 0 | 0 | 0 | 0 | 1 |
| C9JP00 | Muscleblind-like protein 1 | MBNL1 | 7,830,800 | 0 | 0 | 0 | 0 | 2,369,000 | 5,461,800 | 1 |
| A0A6I8PIN8 | F-actin-capping protein subunit beta | CAPZB | 7,662,300 | 0 | 0 | 6,785,600 | 0 | 876,710 | 0 | 1 |
| O95205 | Muscleblind-like protein 2 | MBLL | 7,485,300 | 0 | 0 | 0 | 0 | 1,647,200 | 5,838,000 | 1 |
| A0A590UK99 | Deleted in malignant brain tumors 1 protein | DMBT1 | 7,481,500 | 1,749,100 | 0 | 0 | 3,277,000 | 0 | 2,455,300 | 1 |
| P01766 | Ig heavy chain V-III region BRO | IGHV3-13 | 6,767,800 | 0 | 0 | 0 | 0 | 4,376,000 | 2,391,900 | 2 |
| J3KSH9 | Integrin beta-4 | ITGB4 | 6,700,000 | 0 | 0 | 0 | 0 | 0 | 6,700,000 | 1 |
| P55268 | Laminin subunit beta-2 | LAMB2 | 6,456,200 | 6,456,200 | 0 | 0 | 0 | 0 | 0 | 2 |
| P47929 | Galectin-7 | LGALS7 | 6,362,600 | 0 | 0 | 1,822,500 | 0 | 0 | 4,540,100 | 1 |
| E9PFG7 | 2-oxoglutarate dehydrogenase, mitochondrial | OGDH | 6,288,400 | 6,288,400 | 0 | 0 | 0 | 0 | 0 | 1 |
| E5RK69 | Annexin;Annexin A6 | ANXA6 | 6,072,900 | 0 | 0 | 0 | 0 | 0 | 6,072,900 | 2 |
| E7EQB2 | Lactotransferrin;Lactoferricin-H;Kaliocin-1;Lactoferr | LTF | 6,032,800 | 0 | 3,762,400 | 2,270,400 | 0 | 0 | 0 | 3 |
| E9PRN7 | 3 beta-hydroxysteroid dehydrogenase/Delta 5-->4-is | HSD3B1 | 5,960,500 | 0 | 5,960,500 | 0 | 0 | 0 | 0 | 1 |
| P17661 | Desmin | DES | 5,939,400 | 0 | 5,939,400 | 0 | 0 | 0 | 0 | 5 |
| A0A087X0S5 | Collagen alpha-1(VI) chain | COL6A1 | 5,903,600 | 5,903,600 | 0 | 0 | 0 | 0 | 0 | 2 |
| P0DP09 | Immunoglobulin kappa variable 1-13 | IGKV1D-13 | 5,730,000 | 0 | 0 | 5,730,000 | 0 | 0 | 0 | 1 |
| P62805 | Histone H4 | HIST1H4A | 5,728,200 | 0 | 2,222,200 | 0 | 0 | 3,506,000 | 0 | 2 |
| Q8WVV4 | Protein POF1B | POF1B | 5,534,300 | 0 | 0 | 0 | 0 | 5,534,300 | 0 | 1 |
| A0A1W2PP22 | Heterogeneous nuclear ribonucleoprotein U | HNRNPU | 5,391,800 | 0 | 0 | 0 | 0 | 0 | 5,391,800 | 1 |
| Q9BW30 | Tubulin polymerization-promoting protein family me | TPPP3 | 5,092,600 | 5,092,600 | 0 | 0 | 0 | 0 | 0 | 1 |
| P08572 | Collagen alpha-2(IV) chain;Canstatin | COL4A2 | 5,034,400 | 5,034,400 | 0 | 0 | 0 | 0 | 0 | 1 |
| A0A075B6K5 | Ig lambda chain V-III region LOI | IGLV3-9 | 5,025,200 | 0 | 0 | 2,660,600 | 0 | 0 | 2,364,600 | 1 |
| H0YJL6 | Ena/VASP-like protein | EVL | 4,998,300 | 0 | 0 | 4,998,300 | 0 | 0 | 0 | 1 |
| P59666 | Neutrophil defensin 3;HP 3-56;Neutrophil defensin 2 | DEFA3 | 4,545,800 | 0 | 0 | 0 | 0 | 4,545,800 | 0 | 1 |
| Q9NQP5 | Coagulation factor XIII A chain | F13A1 | 4,473,400 | 4,473,400 | 0 | 0 | 0 | 0 | 0 | 2 |
| E9PS23 | Cofilin-1 | CFL1 | 4,289,600 | 0 | 2,251,300 | 0 | 2,038,300 | 0 | 0 | 2 |
| B4DU11 | Estradiol 17-beta-dehydrogenase 1 | HSD17B1 | 4,274,500 | 0 | 2,367,500 | 1,907,000 | 0 | 0 | 0 | 1 |
| C9J9S3 | Serine/threonine-protein phosphatase;Serine/threon | PPP1CB | 4,214,000 | 0 | 0 | 1,916,400 | 0 | 0 | 2,297,500 | 1 |
| C9JHS9 | Vigilin | HDLBP | 4,198,300 | 0 | 0 | 0 | 1,536,900 | 0 | 2,661,400 | 1 |
| P05187 | Alkaline phosphatase, placental type;Alkaline phosp | ALPP | 4,169,700 | 0 | 0 | 0 | 0 | 0 | 4,169,700 | 2 |
| F8WEW2 | Actin-related protein 3 | ACTR3 | 4,132,700 | 0 | 4,132,700 | 0 | 0 | 0 | 0 | 1 |
| Q9ULV0 | Unconventional myosin-Vb | MYO5B | 4,073,000 | 2,220,100 | 0 | 0 | 1,853,000 | 0 | 0 | 2 |
| J3QSA3 | Ubiquitin-40S ribosomal protein S27a;Ubiquitin;40S | UBB | 4,045,100 | 1,288,700 | 0 | 0 | 0 | 0 | 2,756,400 | 1 |
| B8ZZ51 | Malate dehydrogenase, cytoplasmic | MDH1 | 4,029,700 | 0 | 0 | 4,029,700 | 0 | 0 | 0 | 1 |
| E9PQ34 | Serpin H1 | SERPINH1 | 4,018,600 | 0 | 4,018,600 | 0 | 0 | 0 | 0 | 1 |
| I3L245 | Nuclear pore complex protein Nup88 | NUP88 | 3,921,000 | 0 | 0 | 0 | 0 | 0 | 3,921,000 | 1 |
| Q13263-2 | Transcription intermediary factor 1-beta | TRIM28 | 3,569,200 | 3,569,200 | 0 | 0 | 0 | 0 | 0 | 1 |
| Q9HD89 | Resistin | RETN | 3,487,400 | 0 | 0 | 2,254,600 | 0 | 1,232,800 | 0 | 1 |
| Q9GZM7-3 | Tubulointerstitial nephritis antigen-like | TINAGL1 | 3,481,600 | 0 | 3,481,600 | 0 | 0 | 0 | 0 | 1 |

| | | | | | | | | | | |
|---|---|---|---|---|---|---|---|---|---|---|
| P27824-3 | Calnexin | CANX | 3,444,400 | 0 | 0 | 0 | 0 | 0 | 3,444,400 | 1 |
| A0A3B3ISA6 | Complement C4-A;Complement C4 beta chain;Com | C4B | 3,381,500 | 0 | 0 | 0 | 0 | 0 | 3,381,500 | 1 |
| A6NCI4-3 | von Willebrand factor A domain-containing protein 3 | VWA3A | 3,185,200 | 0 | 0 | 0 | 3,185,200 | 0 | 0 | 1 |
| Q8WV48-5 | Coiled-coil domain-containing protein 107 | CCDC107 | 3,057,000 | 0 | 0 | 3,057,000 | 0 | 0 | 0 | 1 |
| Q00013-2 | 55 kDa erythrocyte membrane protein | MPP1 | 2,911,300 | 0 | 2,911,300 | 0 | 0 | 0 | 0 | 1 |
| Q14677 | Clathrin interactor 1 | CLINT1 | 2,901,400 | 0 | 0 | 0 | 0 | 2,901,400 | 0 | 1 |
| A6NHR2 | 39S ribosomal protein L37, mitochondrial | MRPL37 | 2,898,500 | 0 | 0 | 0 | 0 | 2,898,500 | 0 | 1 |
| P82663-3 | 8S ribosomal protein S25, mitochondrial | MRPS25 | 2,580,800 | 0 | 0 | 0 | 0 | 0 | 2,580,800 | 1 |
| H7C4C8 | T-complex protein 1 subunit theta | CCT8 | 2,560,000 | 0 | 2,560,000 | 0 | 0 | 0 | 0 | 1 |
| P16401 | Histone H1.5 | HIST1H1B | 2,527,600 | 0 | 0 | 2,527,600 | 0 | 0 | 0 | 1 |
| A0A3B3ITI4 | Stress-70 protein, mitochondrial | HSPA9 | 2,413,000 | 2,413,000 | 0 | 0 | 0 | 0 | 0 | 1 |
| Q8TE68 | Epidermal growth factor receptor kinase substrate 8 | EPS8L1 | 2,165,700 | 2,165,700 | 0 | 0 | 0 | 0 | 0 | 1 |
| Q8TDL5 | BPI fold-containing family B member 1 | BPIFB1 | 2,146,600 | 0 | 0 | 0 | 0 | 1,170,700 | 975,880 | 1 |
| P31146 | Coronin-1A | CORO1A | 2,123,800 | 0 | 0 | 0 | 0 | 2,123,800 | 0 | 1 |
| E9PSE0 | MAP kinase-interacting S/T-protein kinase 1 | MKNK1 | 1,976,500 | 0 | 0 | 0 | 1,976,500 | 0 | 0 | 1 |
| A0A1X7SBZ2 | Probable ATP-dependent RNA helicase DDX17 | DDX17 | 1,903,300 | 0 | 0 | 0 | 1,903,300 | 0 | 0 | 1 |
| M0R1B5 | Acetolactate synthase-like protein | ILVBL | 1,702,000 | 0 | 1,702,000 | 0 | 0 | 0 | 0 | 1 |
| M0QX10 | Nuclear pore glycoprotein p62 | NUP62 | 1,380,100 | 0 | 0 | 0 | 0 | 0 | 1,380,100 | 1 |
| P09525-2 | Annexin A4 | ANXA4 | 1,341,500 | 0 | 0 | 1,341,500 | 0 | 0 | 0 | 1 |
| O15400-2 | Syntaxin-7 | STX7 | 1,329,200 | 1,329,200 | 0 | 0 | 0 | 0 | 0 | 1 |
| H3BS21 | Haptoglobin;Haptoglobin alpha chain;Haptoglobin b | HP | 1,311,500 | 0 | 1,311,500 | 0 | 0 | 0 | 0 | 2 |
| U3KQK0 | Histone H2B;Histone H2B type 1-L;Histone H2B typ | HIST1H2BN | 1,266,700 | 0 | 1,266,700 | 0 | 0 | 0 | 0 | 1 |
| K7EJ44 | Profilin-1 | PFN1 | 1,249,400 | 0 | 0 | 0 | 0 | 1,249,400 | 0 | 1 |
| K7EK06 | Phenylalanine--tRNA ligase alpha subunit | FARSA | 1,246,700 | 0 | 1,246,700 | 0 | 0 | 0 | 0 | 1 |
| P10412 | Histone H1.4 | HIST1H1E | 1,033,200 | 0 | 0 | 1,033,200 | 0 | 0 | 0 | 3 |
| A0A087WUX6 | Proteasomal ubiquitin receptor ADRM1 | ADRM1 | 993,900 | 0 | 993,900 | 0 | 0 | 0 | 0 | 1 |
| B1AUU8 | Epidermal growth factor receptor substrate 15 | EPS15 | 797,470 | 797,470 | 0 | 0 | 0 | 0 | 0 | 1 |
| O76041 | Nebulette | NEBL | 651,720 | 0 | 0 | 0 | 0 | 0 | 651,720 | 1 |

**Supplemental Table 4**
**Protein levels of MDMX (A), PICALM (B), OT-R (C) and V1aR (D) in 44 placental extracts.**
MDMX and PICALM values are relative to extract Q1, and OT-R and V1aR values relative to sample V1. Replicate and mean values are shown.

**A**

MDMX

| Patient | % of Q1 | | | | | | | MEAN |
|---|---|---|---|---|---|---|---|---|
| H1 | | 128.4 | 116.9 | 116.5 | *78.2* | 116.9 | 141.1 | 116.3 |
| H2 | | | 19.1 | 60.8 | 32.5 | 18.9 | 16.0 | 29.5 |
| H3 | | | 8.3 | 9.0 | 26.3 | 17.4 | 4.2 | 13.0 |
| I-1 | | 128.8 | 209.0 | 114.6 | 222.2 | 102.8 | 146.1 | 153.9 |
| I-2 | | | | 57.1 | 75.4 | 106.7 | 51.6 | 72.7 |
| I-3 | | 123.6 | 72.5 | 77.1 | 84.1 | 37.8 | 71.7 | 77.8 |
| I-4 | 136.6 | 88.3 | 135.7 | 81.5 | 58.6 | 133.1 | 103.0 | 94.1 |
| J-1 | | | | | 118.1 | 130.2 | 119.1 | 122.5 |
| J-2 | | | | | 92.9 | 56.5 | 58.9 | 69.5 |
| J-3 | | | | | 84.0 | 60.8 | 66.2 | 70.3 |
| L1 | | | 46.6 | 61.1 | 72.0 | 35.8 | 41.9 | 51.5 |
| L2 | | | | | 46.3 | 55.2 | 31.0 | 44.2 |
| L3 | | | | | 72.1 | 69.1 | 47.5 | 62.9 |
| L4 | | | | 11.9 | 23.9 | 16.2 | 8.9 | 16.3 |
| M1 | | | | 57.2 | 57.5 | 29.7 | 62.4 | 51.7 |
| M3 | | | | | | 76.8 | 37.2 | 57.0 |
| M4 | | | | 60.1 | 40.8 | 42.1 | 68.7 | 52.9 |
| N1 | | 60.1 | 40.8 | 42.1 | 68.7 | 101.6 | 101.6 | 115.5 |
| N2 | | 10.2 | 31.0 | 45.5 | 58.0 | 58.2 | 6.6 | 34.9 |
| N3 | | | 75.3 | 167.8 | 85.8 | 80.4 | 68.0 | 95.5 |
| N4 | | | | 55.9 | 52.5 | 60.4 | 48.7 | 54.4 |
| O1 | | | 57.5 | 34.5 | 46.1 | 101.6 | 49.8 | 57.9 |
| O2 | | | | 78.4 | 82.8 | 34.4 | 85.6 | 70.3 |
| O3 | | 77.3 | 58.3 | 22.3 | 42.5 | 11.0 | 8.6 | 36.7 |
| O4 | | | 72.9 | 24.9 | 34.5 | 25.4 | 111.4 | 53.8 |
| O5 | | | | | 244.4 | 229.0 | 145.1 | 206.1 |
| O7 | | | | | 167.2 | 179.6 | 163.3 | 170.0 |
| Q1 | 100.0 | 100.0 | 100.0 | 100.0 | 100.0 | 100.0 | 100.0 | 100.0 |
| Q2 | | 199.7 | 169.8 | 211.0 | 205.4 | 156.5 | 144.9 | 181.2 |
| Q3 | | 90.9 | 90.1 | 76.0 | 75.8 | 74.2 | 88.5 | 82.6 |
| Q4 | | 50.5 | 86.2 | 28.6 | 45.5 | 62.7 | 31.5 | 50.8 |
| R3 | | 28.2 | 99.6 | 57.5 | 20.0 | 60.2 | 77.0 | 54.5 |
| R4 | | | 126.7 | 116.9 | 93.8 | 58.6 | 109.0 | 101.3 |
| R5 | | | | | 33.6 | 79.8 | 48.8 | 54.1 |
| S1 | | | | | 207.4 | 128.9 | 151.4 | 162.6 |
| S2 | | | | | 202.5 | 107.1 | 110.4 | 140.0 |
| T1 | | | | 124.8 | 161.9 | 155.5 | 124.5 | 141.7 |
| T2 | | | | | 115.0 | 91.3 | 82.9 | 96.4 |
| U1 | | | | | 119.7 | 87.7 | 103.7 | 103.7 |
| U2 | | | | | 163.0 | 175.0 | 122.9 | 153.6 |
| V1 | | | | | 75.4 | 71.1 | 21.0 | 55.9 |
| V2 | | | | | 92.8 | 72.9 | 99.0 | 88.2 |
| W1 | | | | | 99.2 | 72.4 | 84.0 | 85.2 |
| W2 | | | | | 261.5 | 197.7 | 173.8 | 211.0 |

**B**

PICALM

| Patient | % of Q1 | | | | | | | MEAN |
|---|---|---|---|---|---|---|---|---|
| H1 | | | | | 21.3 | 31.8 | 34.7 | 29.3 |
| H2 | | | | | 32.8 | 31.2 | 55.6 | 39.9 |
| H3 | | | | 126.3 | 114.0 | 164.0 | 120.6 | 131.2 |
| I-1 | | | | | | 59.9 | 77.3 | 68.6 |
| I-2 | | | | | | 64.9 | 78.8 | 71.8 |
| I-3 | | | | | 31.5 | 28.4 | 10.5 | 23.4 |
| I-4 | | | 15.7 | 14.2 | 2.0 | 7.1 | 2.9 | 8.4 |
| J-1 | 85.4 | 122.8 | 105.8 | 72.0 | 126.2 | 127.1 | 103.0 | 106.0 |
| J-2 | | | | 30.9 | 21.9 | 21.6 | 41.0 | 28.8 |
| J-3 | | | 93.6 | 118.5 | 113.6 | 166.7 | 101.5 | 118.8 |
| L1 | | | | 95.2 | 122.6 | 227.4 | 127.2 | 143.1 |
| L2 | | | | | 60.2 | 58.9 | 91.8 | 70.3 |
| L3 | | | | 100.2 | 102.2 | 142.1 | 94.5 | 109.7 |
| L4 | | | | | 77.4 | 92.1 | 115.4 | 95.0 |
| M1 | | | | 167.9 | 156.1 | 131.4 | 159.8 | 153.8 |
| M3 | | | | | 4.6 | 4.3 | 4.2 | 4.4 |
| M4 | | | | | 106.1 | 78.3 | 104.0 | 96.1 |
| N1 | | | | | 42.5 | 34.5 | 80.1 | 52.4 |
| N2 | | | | 50.7 | 49.6 | 87.1 | 62.5 | 62.5 |
| N3 | | | | | 72.7 | 69.7 | 77.3 | 73.2 |
| N4 | | | | 45.2 | 42.4 | 43.5 | 51.3 | 45.6 |
| O1 | | | | 41.5 | 43.8 | 35.3 | 11.4 | 33.0 |
| O2 | | | | 96.6 | 42.9 | 109.5 | 117.3 | 91.6 |
| O3 | | | | 53.7 | 66.5 | 45.6 | 151.3 | 79.3 |
| O4 | | | | 157.1 | 63.8 | 54.9 | 188.3 | 116.0 |
| O5 | | | | | | 153.7 | 134.3 | 144.0 |
| O7 | | | | | | 142.0 | 216.5 | 179.2 |
| Q1 | 100.0 | 100.0 | 100.0 | 100.0 | 100.0 | 100.0 | 100.0 | 100.0 |
| Q2 | | | 166.3 | 186.0 | 176.6 | 138.0 | 140.5 | 161.5 |
| Q3 | | | | | 115.3 | 138.5 | 138.3 | 130.7 |
| Q4 | | | | | | 198.8 | 188.6 | 193.7 |
| R3 | | | | | 95.8 | 208.0 | 244.3 | 182.7 |
| R4 | | | | | 83.7 | 64.9 | 42.0 | 63.5 |
| R5 | | | | | | 205.3 | 274.9 | 240.1 |
| S1 | | | | 150.9 | 115.3 | 97.3 | 186.9 | 137.6 |
| S2 | | | | 192.9 | 181.9 | 233.0 | 216.2 | 206.0 |
| T1 | | | | | | 173.3 | 169.2 | 171.3 |
| T2 | | | | 226.5 | 181.1 | 130.8 | 219.4 | 189.5 |
| U1 | | | | | 104.2 | 165.3 | 217.6 | 162.3 |
| U2 | | | | | 119.8 | 122.6 | 218.0 | 153.5 |
| V1 | | | | | 147.0 | 207.3 | 288.0 | 214.1 |
| V2 | | | | | 225.3 | 215.0 | 251.0 | 230.4 |
| W1 | | | | 129.3 | 200.6 | 263.6 | 152.0 | 186.4 |
| W2 | | | | | 125.9 | 125.7 | 184.5 | 145.4 |

**C**

OT-R

| Patient | % of V1 | | | | | | MEAN |
|---|---|---|---|---|---|---|---|
| H1 | | | | 31.9 | 32.6 | 34.9 | 33.1 |
| H2 | | | | 44.6 | 47.5 | *54.3* | 48.8 |
| H3 | | | 52.7 | 53.5 | *66.5* | 64.1 | 59.2 |
| I-1 | | | | 45.9 | 44.5 | 43.4 | 44.6 |
| I-2 | 56.0 | 64.8 | 43.0 | 40.5 | *51.5* | *35.1* | 48.5 |
| I-3 | | | 43.3 | 42.7 | 29.3 | 32.2 | 36.9 |
| I-4 | | | | 24.3 | 24.4 | 18.2 | 22.3 |
| J-1 | | | 78.0 | *66.2* | *80.9* | *83.8* | 77.2 |
| J-2 | | | | 64.8 | 55.1 | *44.8* | 54.9 |
| J-3 | | 74.2 | 64.0 | 62.4 | 73.1 | *87.1* | 72.2 |
| L1 | | | | | 85.6 | *77.4* | 81.5 |
| L2 | | | 84.2 | 80.5 | 86.5 | *93.2* | 86.1 |
| L3 | | | 77.8 | 72.1 | 61.6 | 81.6 | 73.3 |
| L4 | | | 79.0 | 70.5 | 82.2 | *82.3* | 78.5 |
| M1 | | | | 43.2 | 37.9 | 41.8 | 41.0 |
| M3 | | | 22.0 | 22.3 | 21.1 | 18.9 | 21.1 |
| M4 | | | | 57.8 | 42.3 | 45.7 | 48.6 |
| N1 | | | 73.6 | 61.4 | 84.1 | 88.0 | 76.8 |
| N2 | | | | 75.7 | 80.6 | 78.6 | 78.3 |
| N3 | | | | 102.2 | 95.9 | 108.0 | 102.0 |
| N4 | | | 56.3 | 55.7 | *76.5* | *55.9* | 61.1 |
| O1 | | | 40.0 | 61.6 | 46.1 | 43.1 | 47.7 |
| O2 | | | | 81.7 | 85.7 | 90.1 | 85.8 |
| O3 | | | 67.0 | 77.0 | 90.8 | 93.7 | 82.1 |
| O4 | | 80.5 | 81.2 | 84.9 | *93.8* | 97.9 | 87.7 |
| O5 | | | 100.2 | 85.9 | 89.7 | 95.0 | 92.7 |
| O7 | | | | 67.1 | 71.1 | *79.1* | 72.4 |
| Q1 | | | 83.6 | 76.2 | 85.0 | 99.4 | 86.1 |
| Q2 | | | 93.4 | 102.4 | 103.9 | 119.0 | 104.7 |
| Q3 | 69.1 | 65.4 | 91.4 | 67.6 | 71.4 | 74.1 | 73.2 |
| Q4 | | | | 77.3 | 70.4 | *82.6* | 76.8 |
| R3 | | | | 117.8 | 101.2 | *107.9* | 109.0 |
| R4 | | | 87.2 | 75.4 | 84.6 | 97.8 | 86.2 |
| R5 | | | 80.3 | 79.4 | 83.4 | *122.1* | 91.3 |
| S1 | | | 108.7 | 99.1 | 116.1 | *118.3* | 110.6 |
| S2 | | 102.9 | 89.2 | 116.9 | 119.2 | 116.5 | 108.9 |
| T1 | | 120.1 | 104.1 | *113.7* | 129.4 | 119.7 | 117.4 |
| T2 | | | 118.7 | *112.9* | *127.8* | 122.0 | 120.4 |
| U1 | | 84.5 | 66.8 | *75.1* | 126.9 | 103.6 | 91.4 |
| U2 | | | 107.0 | 101.2 | *117.9* | 112.0 | 109.5 |
| V1 | 100.0 | 100.0 | 100.0 | 100.0 | 100.0 | 100.0 | 100.0 |
| V2 | | 111.0 | 100.6 | 104.7 | *117.9* | 114.4 | 109.7 |
| W1 | | | 102.0 | 113.8 | 121.5 | *118.2* | 113.9 |
| W2 | | 92.1 | 107.5 | 101.6 | *115.0* | 96.9 | 102.6 |

**D**

V1aR

| Patient | % of V1 | | | | | | MEAN |
|---|---|---|---|---|---|---|---|
| H1 | | | | | 27.0 | 46.7 | 36.9 |
| H2 | | | | 51.5 | 62.7 | 70.4 | 61.5 |
| H3 | | | | | 73.0 | 73.8 | 73.4 |
| I-1 | | | | 77.8 | 61.7 | 72.6 | 70.7 |
| I-2 | | | | | 51.4 | 56.5 | 53.9 |
| I-3 | | | | | 58.5 | 69.7 | 64.1 |
| I-4 | | | | | 56.4 | 67.2 | 61.8 |
| J-1 | | | 41.2 | 43.3 | 54.9 | 67.6 | 51.7 |
| J-2 | | | | | 55.3 | 42.6 | 48.9 |
| J-3 | | | | 56.8 | 61.7 | 75.2 | 64.5 |
| L1 | | | 43.6 | 57.5 | 63.6 | 67.0 | 57.9 |
| L2 | | | | 80.4 | 76.0 | 91.4 | 82.6 |
| L3 | | | | 87.9 | 87.8 | 98.6 | 91.5 |
| L4 | | | | 87.2 | 68.3 | 75.9 | 77.1 |
| M1 | | | | 76.4 | 90.0 | 87.7 | 84.7 |
| M3 | | | | 118.5 | 80.7 | 108.1 | 102.4 |
| M4 | | | 43.9 | 60.7 | 66.4 | 63.1 | 58.5 |
| N1 | | | | 67.4 | 79.6 | 76.5 | 74.5 |
| N2 | | | 65.5 | 58.4 | 72.5 | 81.7 | 69.5 |
| N3 | | | | 71.8 | 70.9 | 85.2 | 75.9 |
| N4 | | 30.3 | 16.9 | 46.6 | 46.1 | 31.8 | 34.3 |
| O1 | | | | 83.8 | 60.3 | 76.4 | 73.5 |
| O2 | | | | 75.0 | 72.3 | 77.4 | 74.9 |
| O3 | | | | 74.5 | 72.2 | 79.3 | 75.3 |
| O4 | | | | 69.9 | 51.4 | 70.2 | 63.8 |
| O5 | | 49.8 | 93.2 | 68.2 | 85.9 | 73.6 | 74.2 |
| O7 | | | | 83.8 | 73.3 | 88.3 | 81.8 |
| Q1 | | | 98.8 | 62.7 | 98.3 | 79.8 | 84.9 |
| Q2 | | 91.1 | 77.3 | 102.2 | 99.7 | 100.8 | 94.2 |
| Q3 | | | | 71.5 | 62.4 | 66.2 | 66.7 |
| Q4 | 68.5 | 60.6 | 96.9 | 78.0 | 73.7 | 82.9 | 76.7 |
| R3 | | 33.1 | 64.9 | 74.7 | 51.4 | 74.6 | 59.7 |
| R4 | | | 98.1 | 81.9 | 92.3 | 103.7 | 94.0 |
| R5 | | | 66.6 | 42.8 | 72.9 | 68.6 | 62.7 |
| S1 | | | | 95.2 | 105.6 | 93.2 | 98.0 |
| S2 | | | | 78.4 | 86.0 | 99.7 | 88.0 |
| T1 | | 129.5 | 91.3 | 102.2 | 77.7 | 100.1 | 100.2 |
| T2 | | 69.6 | 73.3 | 93.1 | 84.7 | 95.1 | 83.2 |
| U1 | | | 63.1 | 51.1 | 77.3 | 65.0 | 64.1 |
| U2 | | | | 78.1 | 79.5 | 87.6 | 81.7 |
| V1 | 100.0 | 100.0 | 100.0 | 100.0 | 100.0 | 100.0 | 100.0 |
| V2 | | | | 93.0 | 98.1 | 107.1 | 99.4 |
| W1 | | | 105.0 | 94.9 | 91.2 | 92.2 | 95.8 |
| W2 | | 111.0 | 62.2 | 103.1 | 99.2 | 75.4 | 90.2 |

**Supplemental Table 5**

**Regression coefficients from univariable analysis of MDMX protein band intensity (relative to internal reference individual Q1)**

| Covariate | Regression Coefficient (95% CI) | P-value |
|---|---|---|
| Age | 1.94 (-0.79, 4.67) | 0.17 |
| Gravidity | 9.66 (3.59, 15.70) | $3x10^{-3}$ |
| Gestational Age | -1.79 (-12.40, 8.86) | 0.74 |
| Body Mass Index in Labor and Delivery Suite | 0.97 (-0.18, 2.13) | 0.11 |
| Race | | |
| White | (REF) | |
| Black | 22.20 (-14.85, 59.26) | 0.24 |
| Asian | -32.49 (-133.62, 68.64) | 0.53 |
| Unknown | -3.74 (-77.21, 69.73) | 0.92 |
| Hispanic | -0.66 (-37.72, 36.39) | 0.97 |
| Preeclampsia | | |
| No | (REF) | |
| Yes | -44.13 (-71.63, -16.64) | $3x10^{-3}$ |
| Diabetes | | |
| No | (REF) | |
| Type II | 47.63 (14.27, 90.00) | $8x10^{-3}$ |
| GDM | 47.91 (5.51, 90.32) | 0.03 |
| Mode of Delivery | | |
| SVD | (REF) | |
| CD prior to onset of Labor | 56.16 (24.13, 88.18) | $1x10^{-3}$ |
| CD after onset of Labor | 34.80 (-4.46, 74.07) | 0.09 |
| VBAC | 5.55 (-85.70, 96.79) | 0.91 |
| Neonate Sex | | |
| Female | (REF) | |
| Male | 5.04 (-24.35, 34.43) | 0.74 |
| Neonatal Weight (g) | 0.03 (0.01, 0.06) | $3x10^{-3}$ |

**Supplemental Table 6**

**Regression coefficients from univariable analysis of PICALM protein band intensity (relative to internal reference individual Q1)**

| Covariate | Regression Coefficient (95% CI) | P-value |
|---|---|---|
| Age | 3.68 (0.28, 7.08) | 0.04 |
| Gravidity | 7.64 (-0.68, 15.95) | 0.08 |
| Gestational Age | 6.88 (-6.62, 20.37) | 0.32 |
| Body Mass Index in Labor and Delivery Suite | 2.06 (0.66, 3.45) | $6x10^{-3}$ |
| Race | | |
| White | (REF) | |
| Black | 17.33 (-24.11, 60.77) | 0.44 |
| Asian | 17.49 (-101.07, 136.05) | 0.77 |
| Unknown | -75.81 (-161.94, 10.32) | 0.09 |
| Hispanic | -46.39 (-89.82, -2.95) | 0.04 |
| Preeclampsia | | |
| No | (REF) | |
| Yes | -35.60 (-73.25, 2.04) | 0.07 |
| Diabetes | | |
| No | (REF) | |
| Type II | 23.28 (-23.57, 70.13) | 0.34 |
| GDM | 38.05 (-21.50, 97.60) | 0.22 |
| Mode of Delivery | | |
| SVD | (REF) | |
| CD prior to onset of Labor | 59.96 (19.24, 100.68) | $6x10^{-3}$ |
| CD after onset of Labor | 5.69 (-44.23, 55.62) | 0.82 |
| VBAC | -50.27 (-166.29, 65.75) | 0.40 |
| Neonate Sex | | |
| Female | (REF) | |
| Male | 19.10 (-18.16, 56.37) | 0.32 |
| Neonatal Weight (g) | 0.03 ($7x10^{-7}$, 0.06) | 0.06 |

**Supplemental Table 7**

**Regression coefficients from univariable analysis of OT-R protein band intensity (relative to internal reference individual T1)**

| Covariate | Regression Coefficient (95% CI) | P-value |
|---|---|---|
| Age | 1.56 (0.13, 2.99) | 0.04 |
| Gravidity | 1.02 (-2.60, 4.64) | 0.58 |
| Gestational Age | 1.73 (-4.00, 7.45) | 0.56 |
| Body Mass Index in Labor and Delivery Suite | 0.55 (-0.07, 1.17) | 0.09 |
| Race | | |
| White | REF | REF |
| Black | -10.77 (-30.15, 8.61) | 0.28 |
| Asian | -6.04 (-58.92, 46.85) | 0.82 |
| Unknown | -27.94 (-66.36, 10.49) | 0.16 |
| Hispanic | -19.53 (-38.90, -0.15) | 0.06 |
| Preeclampsia | | |
| No | REF | REF |
| Yes | -15.28 (-31.12, 0.54) | 0.07 |
| Diabetes | | |
| No | REF | REF |
| Type 2 | 24.16 (5.47, 42.85) | 0.02 |
| GDM | 15.32 (-8.44, 39.07) | 0.21 |
| Mode of Delivery | | |
| SVD | REF | REF |
| CD prior to onset of Labor | 21.82 (4.09, 39.55) | 0.02 |
| CD after onset of Labor | 0.55 (-21.19, 22.29) | 0.96 |
| VBAC | -19.308 (-69.60, 31.44) | 0.46 |
| Neonatal Sex | | |
| Female | REF | REF |
| Male | 8.56 (-7.11, 24.23) | 0.29 |
| Neonatal Weight (g) | 0.01 ($8.36 \times 10^{-4}$, 0.03) | 0.04 |

**Supplemental Table 8**

**Regression coefficients from univariable analysis of V1aR protein band intensity (relative to internal reference individual T1)**

| Covariate | Regression Coefficient (95% CI) | P-value |
|---|---|---|
| Age | 0.63 (-0.30, 1.56) | 0.19 |
| Gravidity | 1.84 (-0.38, 4.07) | 0.11 |
| Gestational Age | -0.72 (-4.34, 2.90) | 0.70 |
| Body Mass Index in Labor and Delivery Suite | 0.20 (-0.20, 0.60) | 0.34 |
| Race | | |
| White | REF | REF |
| Black | 7.77 (-4.51, 20.04) | 0.22 |
| Asian | -15.55 (-49.05, 17.94) | 0.37 |
| Unknown | -13.74 (-38.07, 10.60) | 0.28 |
| Hispanic | -0.24 (-12.52, 12.03) | 0.97 |
| Preeclampsia | | |
| No | REF | REF |
| Yes | -5.17 (-15.46, 5.12) | 0.33 |
| Diabetes | | |
| No | REF | REF |
| Type 2 | 11.84 (-0.34, 24.03) | 0.06 |
| GDM | 7.67 (-7.82, 23.16) | 0.34 |
| Mode of Delivery | | |
| SVD | REF | REF |
| CD prior to onset of Labor | 20.36 (10.58, 30.13) | $6x10^{-3}$ |
| CD after onset of Labor | -1.05 (-13.04, 10.93) | 0.86 |
| VBAC | -9.60 (-18.26, 37.44) | 0.50 |
| Neonatal Sex | | |
| Female | REF | REF |
| Male | -4.53 (-14.46, 5.40) | 0.38 |
| Neonatal Weight (g) | $5x10^{-3}$ ($-3.00x10^{-3}$, 0.013) | 0.23 |

**Supplemental Table 9**

**Regression coefficients from multivariable analysis of MDMX protein band intensity (relative to internal reference individual Q1)**

| Covariate | Regression Coefficient (95% CI) | P-value |
|---|---|---|
| Gravidity | 7.19 (1.24, 13.14) | 0.02 |
| Preeclampsia | | |
| No | (REF) | |
| Yes | -40.61 (-66.18, -15.04) | $3x10^{-3}$ |
| Diabetes | | |
| No | (REF) | |
| Type II | 20.10 (-16.80, 57.00) | 0.29 |
| GDM | 31.20 (-8.81, 71.22) | 0.14 |
| Mode of Delivery | | |
| SVD | (REF) | |
| CD prior to onset of Labor | 9.05 (-26.72, 44.83) | 0.62 |
| CD after onset of Labor | 27.22 (-8.08, 62.52) | 0.14 |
| VBAC | -15.92 (-93.98, 62.13) | 0.69 |
| Neonatal Weight (g) | 0.01 (-0.01, 0.04) | 0.37 |

**Supplemental Table 10**

**Regression coefficients from multivariable analysis of PICALM protein band intensity (relative to internal reference individual Q1)**

| Covariate | Regression Coefficient (95% CI) | P-value |
|---|---|---|
| Age | 1.73 (-1.64, 5.09) | 0.32 |
| Body Mass Index in Labor and Delivery Suite | 1.35 (-0.07, 2.77) | 0.07 |
| Mode of Delivery | | |
| SVD | (REF) | |
| CD prior to onset of Labor | 40.00 (-3.85, 83.85) | 0.08 |
| CD after onset of Labor | 0.82 (-48.29, 49.93) | 0.97 |
| VBAC | -37.87 (-151.23, 75.48) | 0.52 |

**Supplemental Table 11**

**Regression coefficients from multivariable analysis of OT-R protein band intensity (relative to internal reference individual T1)**

| Covariate | Regression Coefficient (95% CI) | P-value |
|---|---|---|
| Age | 0.71 (-0.83, 2.35) | 0.39 |
| Diabetes | | |
| No | REF | REF |
| Type 2 | 14.0 (-11.14, 37.70) | 0.27 |
| GDM | 5.64 (-21.43, 32.00) | 0.68 |
| Mode of Delivery | | |
| SVD | REF | REF |
| CD prior to onset of Labor | 13.25 (-7.83, 34.33) | 0.23 |
| CD after onset of Labor | -1.04 (-23.92, 21.84) | 0.93 |
| VBAC | -0.17 (-68.44, 34.32) | 0.52 |
| Neonatal Weight (g) | $4.05 \times 10^{-4}$ (-0.02, 0.02) | 0.96 |

**Supplemental Table 12**

**Regression coefficients from bootstrapped replicates of univariable analysis of MDMX protein band intensity (relative to internal reference individual Q1)**

| Covariate | (95% quantiles of point estimate from bootstrapped replicates) | Percentage of replicates <0.05 (95% quantiles of P-value) |
|---|---|---|
| Age | (0.43, 2.90) | 1.7% (0.05, 0.77) |
| Gravidity | (6.82, 12.15) | 95.5% (8x10$^{-4}$, 0.06) |
| Gestational Age | (-6.88, 1.78) | 0% (0.25, 0.97) |
| Body Mass Index in Labor and Delivery Suite | (0.60, 1.50) | 12.6% (0.03, 0.34) |
| Race | | |
| White | (REF) | |
| Black | (5.10, 39.4) | 0.9% (0.07, 0.80) |
| Asian | (-53.55, -6.43) | 0% (0.33, 0.91) |
| Unknown | (-30.86, 17.09) | 0% (0.46, 0.99) |
| Hispanic | (-19.69, 13.18) | 0% (0.32, 0.99) |
| Preeclampsia | | |
| No | (REF) | |
| Yes | (-54.91, -28.15) | 91.2% (9x10$^{-4}$, 0.08) |
| Diabetes | | |
| No | (REF) | |
| Type II | (24.47, 60.41) | 60% (2x10$^{-3}$, 0.23) |
| GDM | (24.83, 67.47) | 44.8% (7x10$^{-3}$,0.30) |
| Mode of Delivery | | |
| SVD | (REF) | |
| CD prior to onset of Labor | (38.96, 67.36) | 98.6% (5x10$^{-4}$, 0.04) |
| CD after onset of Labor | (17.16, 55.75) | 15.5% (0.18, 0.46) |
| VBAC | (-25.22, 55.19) | 0% (0.28, 0.99) |
| Neonate Sex | | |
| Female | (REF) | |
| Male | (-10.62, 15.81) | 0% (0.32, 0.99) |
| Neonatal Weight (g) | (0.02, 0.05) | 85.8% (3x10$^{-4}$, 0.14) |

**Supplemental Table 13**

**Regression coefficients from bootstrapped replicates of univariable analysis of PICALM protein band intensity (relative to internal reference individual Q1)**

| Covariate | (95% quantiles of point estimate from bootstrapped replicates) | Percentage of replicates <0.05 (95% quantiles of P-value) |
|---|---|---|
| Age | (2.19, 5.17) | 41% ($7.1x10^{-3}$, 0.27) |
| Gravidity | (3.42, 11.80) | 22% (0.01, 0.46) |
| Gestational Age | (1.82, 11.72) | 0.1% (0.11, 0.82) |
| Body Mass Index in Labor and Delivery Suite | (1.41, 2.66) | 90% ($8x10^{-4}$, 0.09) |
| Race | | |
| White | (REF) | |
| Black | (-7.10, 40.07) | 0% (0.12, 0.97) |
| Asian | (-40.69,111.00) | 0% (0.9, 0.99) |
| Unknown | (-96.07, -53.30) | 2% (0.05, 0.28) |
| Hispanic | (-64.85, -29.39) | 40% (0.01, 0.24) |
| Preeclampsia | | |
| No | (REF) | |
| Yes | (-52.30, -18.88) | 20.3% (0.01, 0.37) |
| Diabetes | | |
| No | (REF) | |
| Type II | (2.82, 45.52) | 0.6% (0.08, 0.91) |
| GDM | (23.35, 56.54) | 0.6% (0.08, 0.51) |
| Mode of Delivery | | |
| SVD | (REF) | |
| CD prior to onset of Labor | (41.39, 79.89) | 88.5% ($9x10^{-4}$, 0.08) |
| CD after onset of Labor | (-17.08, 31.02) | 0% (0.26, 0.99) |
| VBAC | (-82.60, -27.18) | 0% (0.22, 0.67) |
| Neonate Sex | | |
| Female | (REF) | |
| Male | (3.42, 35.19) | 0.3% (0.10, 0.84) |
| Neonatal Weight (g) | (0.01, 0.05) | 35.1% ($3x10^{-3}$, 0.40) |

**Supplemental Table 14**

**Regression coefficients from bootstrapped replicates of univariable analysis of OT-R protein band intensity (relative to internal reference individual T1)**

| Covariate | (95% quantiles of point estimate from bootstrapped replicates) | Percentage of replicates <0.05 (95% quantiles of P-value) |
|---|---|---|
| Age | (1.17, 2.01) | 54.7% (0.01, 0.14) |
| Gravidity | (0.15, 2.08) | 0% (0.29, 0.93) |
| Gestational Age | (-0.00, 3.40) | 0% (0.26, 0.96) |
| Body Mass Index in Labor and Delivery Suite | (0.34, 0.77) | 15% (0.02, 0.30) |
| Race | | |
| White | REF | REF |
| Black | (-17.36, -3.71) | 0% (0.09, 0.74) |
| Asian | (-13.48, 1.09) | 0% (0.62, 0.99) |
| Unknown | (-33.74, -21.97) | 0% (0.10, 0.30) |
| Hispanic | (-24.88, -14.18) | 32% (0.02, 0.18) |
| Preeclampsia | | |
| No | REF | REF |
| Yes | (-20.16, -10.62) | 24% (0.02, 0.22) |
| Diabetes | | |
| No | REF | REF |
| Type 2 | (17.21, 31.63) | 84% (0.00, 0.10) |
| GDM | (8.96, 22.63) | 1% (0.07, 0.48) |
| Mode of Delivery | | |
| SVD | REF | REF |
| CD prior to onset of Labor | (16.54, 27.71) | 83% (0.01, 0.09) |
| CD after onset of Labor | (-5.28, 6.19) | 0% (0.57, 0.99) |
| VBAC | (-29.34, -2.46) | 0% (0.27, 0.93) |
| Neonatal Sex | | |
| Female | REF | REF |
| Male | (4.20, 13.13) | 0% (0.11, 0.62) |
| Neonatal Weight (g) | (0.01, 0.02) | 49% (0.01, 0.17) |

**Supplemental Table 15**

**Regression coefficients from bootstrapped replicates of univariable analysis of V1aR protein band intensity (relative to internal reference individual T1)**

| Covariate | (95% quantiles of point estimate from bootstrapped replicates) | Percentage of replicates <0.05 (95% quantiles of P-value) |
|---|---|---|
| Age | (0.17, 1.12) | 6% (0.03, 0.74) |
| Gravidity | (0.48, 3.04) | 14% (0.02, 0.71) |
| Gestational Age | (-2.20, 0.98) | 0% (0.30, 0.98) |
| Body Mass Index in Labor and Delivery Suite | (-0.03, 0.43) | 2% (0.07, 0.93) |
| Race | | |
| White | REF | REF |
| Black | (1.07, 14.58) | 3% (0.04, 0.88) |
| Asian | (-32.74, -3.26) | 0% (0.09, 0.87) |
| Unknown | (-24.28, -1.85) | 0% (0.09, 0.89) |
| Hispanic | (-7.10, 7.06) | 0% (0.26, 0.99) |
| Preeclampsia | | |
| No | REF | REF |
| Yes | (-11.01, 0.40) | 2% (0.07, 0.94) |
| Diabetes | | |
| No | REF | REF |
| Type 2 | (5.78, 19.09) | 23% ($9x10^{-3}$, 0.41) |
| GDM | (-4.01, 18.40) | 4% (0.04, 0.95) |
| Mode of Delivery | | |
| SVD | REF | REF |
| CD prior to onset of Labor | (14.50, 26.60) | 100% ($4x10^{-5}$, $2x10^{-2}$) |
| CD after onset of Labor | (-7.93, 5.70) | 0% (0.26, 0.99) |
| VBAC | (-7.09, 23.62) | 0% (0.16, 0.97) |
| Neonatal Sex | | |
| Female | REF | REF |
| Male | (-9.92, 0.86) | 0% (0.11, 0.93) |
| Neonatal Weight (g) | ($1.0x 10^{-3}$, $9.0x10^{-3}$) | 2% (0.05, 0.82) |

**Supplemental Table 16**

**Regression coefficients from bootstrapped replicates of multivariable analysis of MDMX protein band intensity (relative to internal reference individual Q1)**

| Covariate | (95% quantiles of point estimate from bootstrapped replicates) | Percentage of replicates <0.05 (95% quantiles of P-value) |
|---|---|---|
| Gravidity | (4.17, 9.81) | 44% (0.01, 0.28) |
| Preeclampsia | | |
| No | REF | |
| Yes | (-54.42, -22.05) | 80% ($1x10^{-3}$, 0.17) |
| Diabetes | | |
| No | (REF) | |
| Type II | (-7.15, 35.26) | 1% (0.10, 0.97) |
| GDM | (7.02, 50.01) | 5% (0.04, 0.78) |
| Mode of Delivery | | |
| SVD | (REF) | |
| CD prior to onset of Labor | (-7.87, 27.66) | 0% (0.22, 0.98) |
| CD after onset of Labor | (7.62, 49.52) | 8% (0.03, 0.69) |
| VBAC | (-45.98, 36.55) | 0% (0.32, 0.86) |
| Neonatal Weight (g) | (-0.002, 0.03) | 2% (0.07, 0.96) |

**Supplemental Table 17**

**Regression coefficients from bootstrapped replicates of multivariable analysis of PICALM protein band intensity (relative to internal reference individual Q1)**

| Covariate | (95% quantiles of point estimate from bootstrapped replicates) | Percentage of replicates <0.05 (95% quantiles of P-value) |
|---|---|---|
| Age | (0.09, 3.35) | 1% (0.07, 0.93) |
| Body Mass Index in Labor and Delivery Suite | (0.55, 2.11) | 26% (0.008, 0.54) |
| Mode of Delivery | | |
| SVD | REF | |
| CD prior to onset of Labor | (17.36, 62.80) | 20% (0.02, 0.48) |
| CD after onset of Labor | (-23.42, 26.23) | 0% (0.31, 0.99) |
| VBAC | (-70.17, -12.72) | 0% (0.29, 0.84) |

**Supplemental Table 18**

**Regression coefficients from bootstrapped replicates of multivariable analysis of OT-R protein band intensity (relative to internal reference individual T1)**

| Covariate | (95% quantiles of point estimate from bootstrapped replicates) | Percentage of replicates <0.05 (95% quantiles of P-value) |
|---|---|---|
| Age | (0.27, 1.17) | 0% (0.17, 0.76) |
| Diabetes | | |
| No | REF | REF |
| Type 2 | (6.09, 21.30) | 0% (0.10, 0.65) |
| GDM | (-1.66, 14.21) | 0% (0.32, 0.99) |
| Mode of Delivery | | |
| SVD | REF | REF |
| CD prior to onset of Labor | (7.33, 19.97) | 0% (0.09, 0.51) |
| CD after onset of Labor | (-6.97, 4.89) | 0% (0.55, 0.99) |
| VBAC | (-27.20, -0.44) | 0% (0.32, 0.98) |
| Neonatal Weight (g) | ($-4x10^{-3}$, $5x10^{-3}$) | 0% (0.52, 0.99) |

**Supplemental Table 19**
**Autoantigens listed by Neiman et al. [52] among the proteins in VEGFR2, MDMX, and PICALM immunoprecipitations (IP).**

| | Uniprot | Gene | Protein name | Autoimmune disease |
|---|---|---|---|---|
| **VEGFR2 IP** | P01023 | A2M | Alpha-2-macroglobulin (Alpha-2-M) | Sjögren's syndrome |
| | E7EVS6 | ACTB | Actin, cytoplasmic 1 (Beta-actin) | Autoimmune hemolytic anemia |
| | P07355 | ANXA2 | Annexin A2 (Annexin II) (Annexin-2) | Antiphospholipid syndrome |
| | P08758 | ANXA5 | Annexin A5 (Anchorin CII) | Antiphospholipid syndrome |
| | P11182 | DBT | Lipoamide acyltransferase BCOADC-E2 | Primary biliary cholangitis |
| | P17661 | DES | Desmin | Sjögren's syndrome |
| | P10515 | DLAT | PDC-E2 | Primary biliary cirrhosis |
| | Q02413 | DSG1 | Desmoglein-1 | Pemphigus foliaceus |
| | P19474 | TRIM21 | E3 ubiquitin-protein ligase TRIM21 | Sjögren's syndrome; Systemic Lupus Erythematosus |
| | Q8WZ42 | TTN | Titin | Myasthenia gravis |
| | P08670 | VIM | Vimentin | Rheumatoid arthritis |
| **MDMX IP** | P01023 | A2M | Alpha-2-macroglobulin (Alpha-2-M) | Sjögren's syndrome |
| | P68133 | ACTA1 | Actin, alpha skeletal muscle | Autoimmune hemolytic anemia; myashenia gravis |
| | P07355 | ANXA2 | Annexin A2 (Annexin II) (Annexin-2) | Antiphospholipid syndrome |
| | P08758 | ANXA5 | Annexin 5 | Antiphospholipid syndrome |
| | P11182 | DBT | Lipoamide acyltransferase BCOADC-E2 | Primary biliary cirrhosis |
| | Q6P0N6 | DST | Dystonin | Bollous pemphigoid |
| | P06733 | ENO1 | α-Enolase | Multiple sclerosis |
| | P35579 | MYH9 | Myosin-9 | Multiple sclerosis |
| | P08559 | PDHA1 | Pyruvate dehydrogenase E1-A type 1 | Primary biliary cholangitis |
| | P21980 | TGM2 | Isopeptidase TGM2 | Celiac's disease |
| | Q08188 | TGM3 | Transglutaminase-3 | Dermatitis herpetiformis |
| | P29401 | TKT | Transketolase | Multiple sclerosis |
| | P19474 | TRIM21 | E3 ubiquitin-protein ligase TRIM21 | Sjögren's syndrome; Systemic Lupus Erythematosus |
| | P18206 | VCL | Vinculin | Myasthenia gravis |
| **PICALM IP** | P01023 | A2M | Alpha-2-macroglobulin (Alpha-2-M) | Sjögren's syndrome |
| | E7EVS6 | ACTB | Actin, cytoplasmic 1 (Beta-actin) | Autoimmune hemolytic anemia |
| | P63261 | ACTG1 | Actin, cytoplasmic 2/γ-actin | Autoimmune hemolytic anemia; myashenia gravis |
| | P63267 | ACTG2 | Alpha-actin-2 | Autoimmune hemolytic anemia; myashenia gravis |
| | P07355 | ANXA2 | Annexin A2 (Annexin II) (Annexin-2) | Antiphospholipid syndrome |
| | P08758 | ANXA5 | Annexin A5 (Anchorin CII) | Antiphospholipid syndrome |
| | P17661 | DES | Desmin | Sjögren's syndrome |
| | P36957 | DLST | 2-oxoglutarate dehydrogenase E2 | Pernicuous anemia |
| | Q08554 | DSC1 | Desmocollin-1 | IgA pemphigus |
| | Q02413 | DSG1 | Desmoglein-1 | Pemphigus foliaceus |
| | Q9Y285 | FARSA | Phenylalanyl-tRNA synthetase α-subunit | Schizophrenia |
| | Q02218 | OGDH | 2-oxoglutarate dehydrogenase E1 | Pernicuous anemia |
| | Q5JPU3 | PDHA1 | Pyruvate dehydrogenase E1-A type 1 | Primary biliary cholangitis |
| | P21980 | TGM2 | Isopeptidase TGM2 | Celiac's disease |
| | P29401 | TKT | Transketolase | Multiple sclerosis |
| | P19474 | TRIM21 | E3 ubiquitin-protein ligase TRIM21 | Sjögren's syndrome; Systemic Lupus Erythematosus |
| | Q13263 | TRIM28 | E3 SUMO-protein ligase TRIM28 | Myositis |
| | Q8WZ42 | TTN | Titin | Myasthenia gravis |
| | P08670 | VIM | Vimentin | Rheumatoid arthritis |

**Supplemental Table 20**
Patient demographics

| Clinical characteristics | Study subjects (n=44) | % of n |
|---|---|---|
| Maternal Age | | |
| 18-34 | 40 | 91% |
| ≥35 | 4 | 9% |
| BMI | | |
| Underweight (<18.5) | 0 | 0% |
| Normal (18.5-24.9) | 3 | 7% |
| Overweight (25-29.9) | 9 | 20% |
| Obesity I (30-34.9) | 12 | 27% |
| Obesity II (35-35.9) | 5 | 11% |
| Extreme Obesity (≥40) | 15 | 34% |
| Parity | | |
| Nulliparous | 11 | 25% |
| Multiparous | 33 | 75% |
| Gestational Age at Delivery | | |
| < 37 weeks | 4 | 9% |
| ≥ 37 weeks | 40 | 91% |
| Race | | |
| White | 17 | 39% |
| African American | 12 | 27% |
| Hispanic | 12 | 27% |
| Other | 3 | 7% |
| Diabetes | 14 | 32% |
| Type 1 | 0 | 0% |
| Type 2 | 9 | 20% |
| Gestational | 5 | 11% |
| Chronically Hypertensive | 6 | 14% |
| Normotensive | 28 | 64% |
| Preeclamptic | 16 | 36% |
| Preeclamptic and Diabetic | 4 | 9% |
